# Reducing Plate Waste in Latvian Schools: Evaluating Interventions to Promote Sustainable Food Consumption Practices

**DOI:** 10.3390/foods14010126

**Published:** 2025-01-04

**Authors:** Jelena Lonska, Sergejs Kodors, Juta Deksne, Lienite Litavniece, Anda Zvaigzne, Inese Silicka, Inta Kotane

**Affiliations:** 1 Research Institute for Business and Social Processes, Faculty of Economics and Management, Rezekne Academy of Technologies, LV-4601 Rezekne, Latvia; juta.deksne@rta.lv (J.D.); lienite.litavniece@rta.lv (L.L.); anda.zvaigzne@rta.lv (A.Z.); inese.silicka@rta.lv (I.S.); inta.kotane@rta.lv (I.K.); 2 Institute of Engineering, Faculty of Engineering, Rezekne Academy of Technologies, LV-4601 Rezekne, Latvia; sergejs.kodors@rta.lv

**Keywords:** food waste, plate waste, school catering, food waste-reducing interventions

## Abstract

Food waste (FW) threatens food security, environmental sustainability, and economic efficiency, with about one-third of global food production lost or wasted. Schools play a crucial role in addressing FW, representing lost resources and missed educational opportunities. The present research assessed three interventions to reduce plate waste (PW) in Rezekne City schools, namely (S1) a plate waste tracker, (S2) an awareness and educational campaign, and (S3) organizational changes, including larger plates, extended lunch breaks, and teacher supervision. Implemented in three schools with a fourth as a control, PW was measured at three intervals, at pre-intervention, short-term, and long-term post-intervention. The PW data analysis utilized two models (day view and class view) and a Wilcoxon signed-rank test. While the plate waste tracker initially reduced PW, sustained impact required continuous reinforcement. The awareness and educational campaign alone proved insufficient, highlighting the need for complex strategies. The organizational changes unexpectedly increased PW, underscoring FW’s complexity. The research has concluded that reducing FW requires tailored and multi-faceted approaches. According to the MOA framework, the school catering model in Rezekne City lacks essential “Opportunities” for effective FW reduction, as students have limited flexibility in portion sizes and food choices, which hinders the interventions’ effectiveness. Future research should explore adaptable FW-reducing interventions suited to specific school contexts.

## 1. Introduction

High FW levels are attracting global attention, and FW reduction is one of the targets within the sustainable development framework developed by the United Nations [1]. Over the last ten years, food loss (FL) and FW have become a global problem. FW is not only an ethical and economic issue but also an environmental one; environmentally, FW contributes to the unnecessary use of resources such as water, energy, and land, which harms our soil, air, and water quality; economically, it represents a significant loss, driving up costs and reducing efficiency in the food supply chain; socially, FW increases food insecurity, as good food is thrown away while many people go hungry [2,3,4,5]. The European policy report on food loss and waste [6] calls for a more unified approach to address these issues. It suggests that reducing FW can play a key role in making our food system more sustainable. This would improve food security and public health, help to restore the environment, protect biodiversity, and maintain the value and quality of food.

Globally, approximately a third of all food produced for human consumption is lost or wasted [2]. According to the UNEP Food Waste Index (2024) [7], around 1.05 billion tons of FW were wasted across three sectors in 2022 (or 132 kg per capita)—60% of which came from households (79 kg per capita), 28% from food services (36 kg per capita, including ISIC 85 sector “Education”, specifically canteens and other places for the preparation and consumption of food associated with educational settings), and 12% from retail (17 kg per capita). This amounts to one-fifth (19%) of food available to consumers being wasted at the retail, food service, and household levels. In high-income countries, the composition of FW varies slightly, at 81 kg per capita in households, 21 kg per capita in food service, and 13 kg per capita in retail.

In the EU, over 58 million tons of FW are generated annually [8], with associated costs estimated at EUR 132 billion [9]. According to the latest EU data, 70% of total FW arises at consumption and retail, with households generating more than half of the total FW in the EU (54%) [8]. Addressing consumer FW is crucial to achieving Sustainable Development Goal 12, Target 12.3, of halving per capita global FW at the retail and consumer level by 2030 [1].

The recent EU strategies [10,11,12,13] include some measures to reduce FW. The European Commission intends to set legally binding FW reduction goals throughout the EU as well as to incorporate food loss and waste prevention targets into other EU policies [12].

Reducing FW requires all food system actors to work together—this is where educational institutions have an important role to play in raising students’ awareness of the importance of preventing and reducing FW. Schools play a crucial role in providing information on healthy and sustainable food consumption, which can help to shape the habits of the new generation, including those related to FW. Some foreign researchers [14] point out that the school catering sector is one of the largest sources of FW at the food service stage, and at the same time, this also provides an opportunity to improve the dietary habits of the population and educate the public about sustainable resource consumption and development, thereby affecting the food system in the future.

The present research focuses on managing the school catering process in four selected schools in Rezekne City (Latvia) that provide free lunches for students. The purpose of this pilot study is to assess the impacts of interventions aimed at reducing the amount of PW in three schools with a fourth as a control. The subject of the research includes both individual PW and discarded served food from common containers after free lunches. The research explores the hypothesis that implementing targeted interventions to reduce PW can effectively decrease the quantity of uneaten food, thereby promoting more sustainable food consumption practices. During the research, multiple pre-intervention and post-intervention PW quantifications were made to statistically test the impacts of interventions on reducing the amount of PW.

This study provides a significant contribution to the field of FW research by focusing on interventions to reduce PW in school canteens—a largely underexplored area in Latvia. Unlike previous studies conducted in countries with buffet-style catering systems, this research addresses the challenges of a partly pre-portioned catering model, which is widely used in Latvian schools. By experimentally testing three targeted interventions—a plate waste tracker, an awareness campaign, and organizational changes—this study highlights the processes of transferring, adapting, and evaluating international best practices within a Latvian context. The findings underscore the importance of tailoring interventions to specific organizational settings, offering practical insights for policymakers and school administrators aiming to reduce FW and foster sustainable consumption habits among students. Moreover, the application of the Motivation–Opportunities–Abilities (MOA) framework provides a structured approach to understanding the behavioral prerequisites of FW, making this research a valuable reference for both academic and practitioner communities seeking scalable solutions to FW challenges in schools.

The structure of this paper is as follows: Section 2 provides an in-depth review of the relevant literature on existing FW-reducing interventions at the FSC consumption level, particularly in school catering. Section 3 outlines the materials and methods employed by the research, focusing on the interventions applied in Rezekne City schools to reduce PW. Section 4 presents a data analysis and the results, detailing the short- and long-term impacts of the interventions across the participating schools. This section also includes statistical tests, including a Wilcoxon signed-rank test used to assess intervention effectiveness. Finally, Section 5 offers a discussion of the findings, while Section 6 concludes the research, addressing potential implications for policy and recommendations for future research on sustainable FW reduction in schools.

## 2. Theoretical Background and a Literature Review

### 2.1. Food Waste at the Consumption Stage

**Figure 1:**
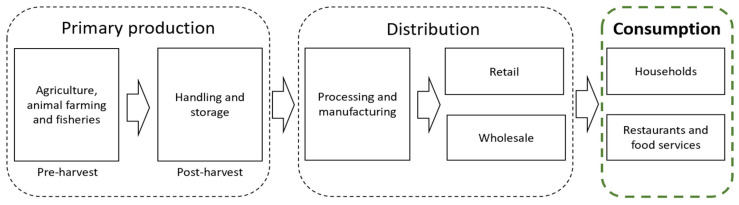
Stages in the FSC at which food might be lost or discarded (based on [17]).

Academics categorize FW based on the stages of waste generation, such as pre- and post-consumer FW [15]. Pre-consumer waste occurs at the food supply chain (FSC) primary production and distribution levels, and pre-consumer FW is often called food loss, while post-consumption waste occurs at the consumption level [16]. This research focuses on an analysis of FW and measures to reduce it at the consumption level (see Figure 1).

Consumers are the primary contributors to FW across the food supply chain in high-income countries, accounting for an estimated 53% [18]. Given that a significant portion of this waste could be avoided, it is evident that there is an urgent need to change consumer behavior [19].

The European Commission, through its Farm to Fork strategy [12] and its broader European Green Deal policy [20], commits to ambitious food systems objectives, which can be achieved by driving a step-wise, learning-focused policy transformation at the global, EU, national, regional, and local levels [21]. Some of the barriers to the reduction in FW are referred to, such as (1) food operators and consumers do not have adequate information on how much they waste (consumers often underestimate the amount of food they waste), nor on the possible options to reduce FW and (2) a lack of willingness of actors to adopt FW-reducing innovations among consumers [22]. As one of the enablers of change, the following solution is mentioned: (1) to improve action design, monitoring, evaluation, and knowledge sharing regarding FW prevention interventions and (2) to integrate FW reduction in school education and professional training, both in the public and private sectors, thus promoting the value of food and working to shift social norms so that wasting food is no longer acceptable, etc. [22].

In addition, the UN Environment Program notes that accurate, traceable, and comparable FW measurement is a key starting point for national FW strategies and policies to deliver the 50% reduction in consumer FW targeted in the 12th SDG Target 12.3 [23]. It is necessary to measure FW as accurately as possible; therefore, weight measurements are considered the gold standard in FW measurements [24].

According to the Latvian Waste Prevention Plan developed by the European Environment Agency, in 2018, the total amount of FW generated in Latvia reached 319 thousand tons, with primary production accounting for 5% (16 thousand tons); processing and manufacturing 37% (117 thousand tons); and trade < 1% (two thousand tons). Most of the FW, i.e., 57% or 185 thousand tons, ended up in municipal solid waste, and a part was mainly discharged by households and food services. As not all producers of FW and surpluses are obliged to report the amount of waste generated, the information summarized above is indicative [25]. Arina et al. [26] estimated that in 2020 in Latvia, 157 thousand tons (or 83 kg per capita) of FW was generated by the household sector, while 11 thousand tons (or almost 6 kg per capita) by restaurants and food services [26].

In 2021 in Latvia, according to Eurostat, the total FW per capita averaged 130 kg, close to the EU average of 131 kg per capita [8]. Table 1 presents the amounts of FW reported by the EU Member States (average amount) and Latvia for the reference year 2021, measured in tons of fresh mass and as a % share of the total amount by sector of activities.

As shown in Table 1, most of the FW is generated at the food consumption stage both in the EU as a whole and in Latvia, i.e., by restaurants and food services, as well as households: in the EU, on average, it is 62.3% or 81.7 kg per capita, while in Latvia, it is 67.3% or 87.8 kg per capita. It could be concluded that the amount of FW reported by Latvia is 6.1 kg higher than the EU average at the food consumption stage. The data clearly point to the need to actively promote FW reduction at the consumer level.

### 2.2. Complexity of School Food Consumption Behavior

Consumer behavior related to FW generated in schools is influenced by a combination of internal and external factors, making it essential to understand the various influences that drive individual decision-making. While Lonska et al. [27] emphasize the role of both exogenous and endogenous factors in shaping school food consumers’ behaviors, it is equally important to explore how these factors interact within broader behavioral frameworks. At the FSC consumption stage, this interplay becomes particularly relevant, as interventions targeting FW reduction must account for these complexities through robust theoretical approaches.

One of the earliest and most frequently applied frameworks is the theory of planned behavior (TPB). However, the TPB primarily focuses on cognitive drivers, treating FW as an intended behavior, which limits its scope [28,29]. To address this limitation, the Motivation–Opportunities–Abilities (MOA) framework has been suggested as a more comprehensive approach to classify the drivers, levers, and interventions related to consumer FW [30,31], which has been used in FW research in both academic and practitioner settings. The MOA framework broadens the analysis beyond cognitive aspects by incorporating the Motivation element, which includes attitudes, intentions, and norms as outlined in the TPB, and adding Opportunities and Abilities elements, which extend the framework beyond cognitive boundaries. Unlike the TPB, the MOA framework views FW not as a solely intended behavior but as an unintended consequence of a series of decisions and behaviors associated with food management practices both inside and outside the home. These practices are influenced by both internal (individual) and external (social and societal) factors [29,32,33,34,35].

Motivation to prevent FW refers to an individual’s willingness to take actions that minimize the occurrence or quantity of FW. Key factors influencing motivation include attitude, awareness, and social norms. Opportunities to prevent FW involve the availability and accessibility of the necessary materials and resources to reduce FW, for instance, time management or a daily schedule, available food infrastructure and technologies, and food policies. Abilities involve the skills and knowledge required to carry out a behavior successfully. The MOA framework emphasizes that in order for consumers to successfully act on a FW reduction, there must not only be a strong motivation to do so but also an absence of barriers that might obstruct their efforts. These barriers often include factors that lead consumers to believe they are incapable of reducing FW. Even if someone is motivated to reduce FW, without the necessary skills or knowledge—such as understanding proper food storage techniques—they might find it difficult to achieve their goals [33,34].

According to the MOA framework, effective behavior change, such as reducing FW, occurs when these three elements—motivation, opportunities, and abilities—are aligned. When all three are present, simple interventions such as informational reminders might be enough to sustain the desired consumer behavior. However, if any of these elements are lacking, more targeted interventions are required. For instance, if motivation is low, strategies such as regulatory incentives, nudging, competition, or social influence campaigns might be necessary. If abilities are lacking, educational campaigns providing practical tips can help. And if opportunities are limited, introducing new products or services could create the necessary prerequisites for behavior change. The MOA framework highlights that achieving and maintaining behavioral change, such as reducing FW, requires addressing all three components. Without a balanced approach, individuals are likely to revert to their previous behaviors once the interventions are removed [32].

**Figure 2:**
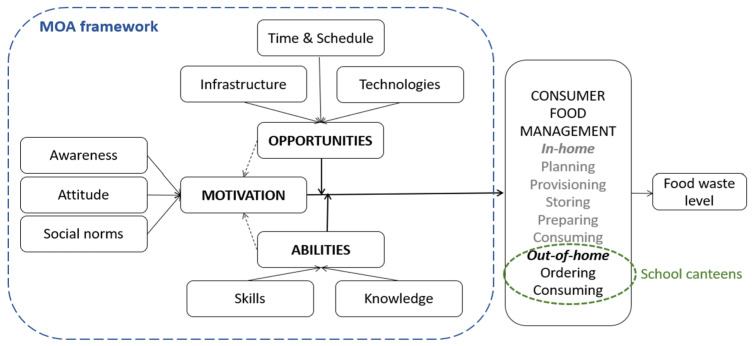
MOA framework and the consumer food management model for in-home and out-of-home consumption (authors’ modification based on [29]).

The MOA framework allows us to analyze in-home and out-of-home consumer food management. It is important for the present research to apply the MOA framework to analyze consumer food management in school canteens (see Figure 2).

As shown in Figure 2, motivation, abilities, and opportunities to engage in FW prevention affect the amount of consumer FW generated. Under the out-of-home consumer food management model, food is being moved from provisioning to consumption, passing (all) intermediate stages. In the case of school catering, students as food consumers can only affect the amount of FW at the ordering/serving and consuming stages, i.e., the differentiation of portion sizes, the choice of a food type, and the eating behavior of students are important at these stages.

### 2.3. Overview of Interventions to Reduce Consumer Food Waste

In European countries, various initiatives or interventions aimed at reducing consumer FW have been launched over the last 10–15 years. Next, a number of EU-level and project reports are reviewed to identify and classify the most commonly implemented interventions to deal with consumer FW in Europe.

Wunder et al. [36], in the policy report on consumer FW “REFRESH: Consumers and Food Waste”, highlight the complexity of consumer FW, influenced by the consumers’ desire for convenience, taste preferences, and cost-saving behaviors such as bulk buying and promotions. It should be noted that the report also covered FW mitigation interventions that can influence food consumer behavior at the retail stage, which, as shown in Figure 1, belong to the FSC distribution level. The authors categorize policy instruments into information campaigns, regulation, economic measures, nudging, and voluntary agreements (see Table 2).

The report concludes that informational and awareness-raising campaigns alone are often ineffective in significantly reducing FW but prompts, skill training, social norm campaigns, and feedback mechanisms show more promise. A systematic, integrated approach involving collaboration with the retail and hospitality sectors and a thorough assessment of the effectiveness of interventions is essential for impactful FW reduction [36].

The European Commission has developed a series of action plans and regulatory measures to deal with FW in the EU. One of the most important steps was the implementation of the ECFWF (European Consumer Food Waste Forum) project [37], which provided practical tools and recommendations to reduce FW at the consumer level. The ECFWF evaluated 78 consumer-level FW measures, revealing the varying effectiveness of different approaches. The interventions evaluated were classified as follows (Table 3):

It is evident that in the EU, FW reduction interventions are being implemented at the macro (local and national government), meso (trade associations, producer groups, NGOs), and micro (entrepreneur and consumer) levels, e.g., by schools or restaurants. The key findings made by the ECFWF highlight that the interventions tailored to local contexts and involving community and stakeholder collaboration are more successful. Disruptions to daily routines promise to reduce household FW, and highly personalized interventions yield positive outcomes, especially if consumers participate voluntarily. However, no single intervention proves universally effective, indicating that a multifaceted strategy combining various interventions is necessary to significantly reduce consumer FW [38].

A literature review conducted by Caldeira et al. [66] showed that there was a notable lack of studies dealing specifically with the evaluation of FW prevention actions. In this report, 91 actions were collected through a survey and individually assessed to test the evaluation framework developed. Most (58) were implemented at the FSC food service and household stages. The classification is presented in Table 4.

This report highlights that effective FW prevention relies on a multifaceted approach at the food consumption stage. The assessment of FW prevention actions revealed that most of the initiatives focused on consumer behavior change, food redistribution, and improving supply chain efficiency. These actions demonstrated varying levels of success, with some effectively reducing FW through awareness campaigns and redistribution efforts, while others highlighted the need for better design and implementation strategies. The analysis underscored the importance of setting clear objectives, monitoring progress, and adapting approaches to local contexts to enhance the effectiveness and sustainability of FW prevention measures at the food consumption stage [66].

Cooperation between all food system actors is essential to reduce FW, with educational institutions playing a key role. By providing information on healthy and sustainable diets, schools can shape the habits of the next generation and affect the future food system, as school meals are one of the largest sources of FW. It is undeniable that responsible food consumption in schools can contribute to reducing FW at the FSC consumption stage; therefore, it is important to identify and classify FW-preventing interventions addressing students’ behavioral change, which could be implemented in schools (see Table 5). FW prevention in school and school canteens could set a positive example for children and young people and inspire them to do the same at home. Understanding the nature of various interventions aimed at reducing FW in schools, policymakers and school administrators could develop and adapt food resource efficiency strategies.

Table 5 presents various effective interventions to reduce FW in schools through behavioral and attitudinal changes among students. Visual nudging interventions, such as posters and signs, can raise students’ awareness of FW problems. Participatory nudging interventions involve students in activities such as FW audits and cooking workshops. Educational nudging interventions integrate food sustainability topics into the curriculum, which can have a positive impact on the students’ food consumption habits. Food choice architecture involves designing and presenting food options to effectively influence students to make more efficient consumption choices, using strategic placement and presentation to reduce waste. Altering the dining environment, e.g., extended lunch breaks or modifying the dining setting and conditions to encourage students to make sustainable food choices and practice responsible consumption, are aimed at creating a more pleasant and quiet dining experience. Feedback ensures continuous improvement. The mentioned multifaceted interventions, tailored to the local context, can significantly reduce FW in schools and promote a culture of sustainability.

It is clear that there is a need for a multi-faceted approach to reducing FW at the FSC consumption stage, especially in schools. The variety of interventions aimed at reducing FW, ranging from visual and participatory nudging to educational activities and environmental change, highlight the complexity and multidimensional nature of effectively addressing FW. By implementing these actions toward sustainability, schools can influence students’ food consumption habits, thus contributing to a culture of responsible consumption among the younger generation.

## 3. Materials and Methods

### 3.1. Scope of Research

This pilot study aimed to evaluate the impacts of interventions designed to reduce PW in three schools with a fourth as a control in Rezekne City, Latvia, to promote smart and responsible food consumption. The underlying hypothesis was that targeted interventions in these schools could effectively lower PW levels, thereby fostering sustainable food consumption practices. To assess the impacts of the interventions, PW quantities were measured multiple times before and after the implementation to provide a statistical basis for evaluating PW reduction.

The total measured weight of PW included uneaten food left on individual plates and discarded food in common bowls and pots following the free lunches provided to students in grades 1–7 in the observed schools (see Section 3.4).

The novelty of our pilot study lies in the fact that no national-level research has previously been conducted in Latvia to verify the effectiveness of interventions made by foreign researchers aimed at reducing FW in the Latvian school ecosystem. This is particularly important, considering that the management of catering services in Latvian schools is significantly different from foreign practices.

Within our previous research study, a comprehensive literature review was performed, and a large number of research studies on factors contributing to PW in schools were reviewed [27]. An experiment by the Swedish University of Agricultural Sciences with the aim of testing interventions related to reducing FW in school catering could be mentioned as the most relevant research on the research problem [74]. The following interventions were examined during the Swedish experiment: (1) tasting spoons; (2) an awareness campaign; (3) a plate waste tracker; (4) a forecasting system; and (5) a reference group. However, the management of catering in Swedish schools and the level of public awareness of responsible food consumption, as well as the socio-economic culture, are significantly different from those in Latvia. For example, in Latvia, self-service (buffet-style) catering is rarely practiced in schools for those schoolchildren whose catering expenses are covered by state/municipal funding, and it is not possible to choose the type of food, as the food is already served following all the dietary guidelines regarding the amount of food served, calories, and nutrients. This means that schoolchildren cannot choose the size of the portion themselves (smaller or larger, depending on the feeling of hunger or age). We can also observe similar differences in many other research studies conducted outside Latvia [14,119,120,121,122,123].

Consequently, a natural question arises as follows: can foreign experience be effectively transferred to Latvia? Based on the fact that no scientific research in this field has been conducted in Latvia to date, we decided to assess how effectively certain interventions examined by the Swedish University of Agricultural Sciences (awareness campaign and plate waste tracker) worked in Latvia. However, given the specifics of the management of catering in Rezekne City schools (there is no buffet-style catering), it was not possible to transfer all interventions proposed by the Swedish colleagues; therefore, the 3rd intervention component (using larger diameter plates for serving food, holding longer lunch breaks, and ensuring the presence of a supervising teacher during the lunch break) was chosen based on the recommendations we proposed in our previously implemented “E-mentor” project [124] after analyzing global best practices. All the interventions proposed were coordinated with the administrations of the selected schools, receiving their support. We also contacted colleagues from the Swedish University of Agricultural Sciences about the possibility of using their plate waste tracker in our research and localizing its functionality for the region of Latvia. After summarizing the above, it could be found that the novelty of our project involves experimentally testing foreign best practices aimed at PW reduction in Latvia, as well as making cross-cultural comparisons of the results, which is essential when continuing to implement interventions in the long term.

### 3.2. Research Methodology

The research intends to use the scientific findings made in our previous research, thereby resulting in the development of a set of recommendations (interventions) for stakeholders to be implemented to reduce the amount of PW in Rezekne City schools [27]. PW accounts for the majority of FW in schools [125]. It should be noted that most of the researchers working on FW analysis focus specifically on PW analysis [121,125,126,127,128,129,130,131,132,133]. Derqui and Fernandez [91] have found that approximately 80% of research in this field directly relates to PW analysis without auditing FW at the whole stage of food consumption, i.e., not considering the FW generated during cooking in the kitchen or the FW from serving lines.

Of the 6 schools in Rezekne City offering free lunches to students in grades 1–7, 4 were selected based on the willingness of school principals to collaborate, ensuring smooth coordination and effective implementation of the interventions. All schools operate under a similar catering model with partly pre-served meals, providing a uniform context for evaluation. The schools represent urban Latvian schools, where free lunches are provided to students in grades 1–7 through state and municipal funding. The student populations in grades 1–7 across the schools are similar, ensuring comparable sample sizes and demographics. While limited to Rezekne City, the findings offer valuable insights for similar school settings across Latvia with analogous catering systems.

During our previous research, we found that there was a need for interventions that could reduce the amount of PW in Rezekne City schools. Thus, we decided to implement several of the proposed interventions in three schools (a test group). One more school participated as a reference group for PW quantification, yet no special interventions aimed at reducing PW were planned. Malefors et al. [74] used the reference group to examine whether the test interventions reduced FW or whether reductions were due to other trends and ambitions that would have happened in any case.

**Figure 3:**
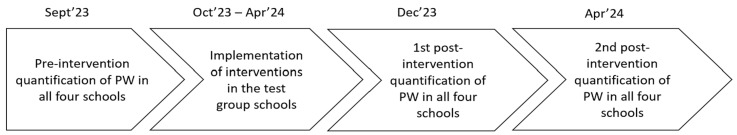
Timeline of the pilot research.

The research comprised the following main steps (see Figure 3):

Statistical analysis was applied to verify the impact of our interventions. The null hypothesis “PW is equal in the pre- and post-intervention periods” was tested. The following laboratory conditions were organized in all four schools: (1) similar classes participated in the survey and (2) a unified menu design was applied in pre- and post-intervention PW measurement weeks. The unified menu for the field study was developed for one working week within our previous research (for details, see Lonska et al. [27]). As a result, the paired method was applied for statistical analysis; for Model 1 (class view), the average PW g/student data were calculated per class. In schools, classes were divided into sections A, B, and C. Therefore, each school had 15–20 pairs for comparison depending on the school, and for Model 2 (day view), the average PW g/student data per day were calculated for each school. Additionally, a comparison between 5 days for PW and g/student for each school was performed to exclude the impact of the menu. A Wilcoxon signed-rank test was performed to test each intervention within both models.

### 3.3. Description of the Implemented Interventions

The following interventions aimed at a reduction in PW were tested: School 1 (S1)—a plate waste tracker; School 2 (S2)—an awareness and educational campaign; and School 3 (S3)—a set of organizational changes, including larger diameter plates used in the can-teen, longer lunch breaks, and the presence of the supervising teacher during the lunch break. The interventions were implemented from 1 October 2023 to 30 April 2024. The capability of the interventions to reduce PW in school canteens was tested against both the baseline before implementation and a reference School 4 (S4), in which no intervention was implemented. The objective was to identify the interventions that could be scaled up so that school canteens can achieve larger-scale reductions in PW necessary for a sustainable food system.

#### 3.3.1. Plate Waste Tracker

**Figure 4:**
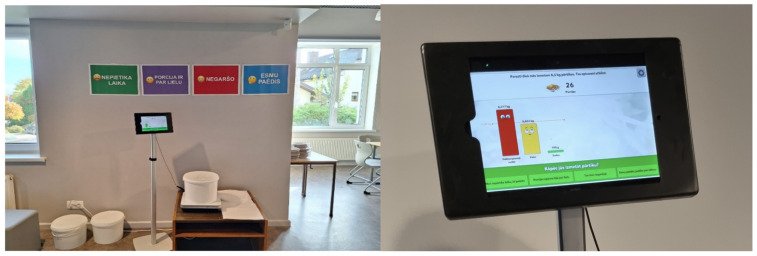
Plate waste tracker installed in the S1 canteen.

As part of the research study in S1, a plate waste tracker was installed (Matomatic AB, Uppsala, Sweden) [134]. The plate waste tracker is a kitchen scale connected to a tablet computer running dedicated software that interacts with canteen visitors, showing them how much food they are wasting and the impact of this waste. The tablet computer allows the canteen visitors to respond to why they wasted food, with some predefined alternatives, such as “I did not have enough time to eat”, “The portion size was too large”, “I did not like it”, and “I am full” [74]. The device was adapted for use in Latvia by installing the Latvian language. However, because only one device was installed at the school, we encountered a situation where long lines of students formed during the lunch break, as they had to dispose of their PW on the tracker scales. Additionally, this process was slowed down by the fact that some primary schoolchildren did not yet read quickly; therefore, providing their feedback took extra time. At the beginning of the intervention, we observed that some students lacked the time to throw away their PW during the lunch break. We solved this problem by hanging the possible reasons for PW, as provided by the tracker, on the wall right next to the tracker. The students could then tell the school personnel operating the device why they did not eat all the food, and the personnel would enter the students’ answers into the tracker (see Figure 4).

#### 3.3.2. Awareness and Educational Campaign

In S2, an intervention to reduce PW was implemented through a combination of awareness and educational campaigns [14,68,71,120,135,136,137]. Preventive measures aimed at reducing FW during the FSC consumption phase emphasize several key approaches to educate consumers and alter behaviors to minimize waste. The approaches include public awareness efforts, educational programs in schools, and waste reduction initiatives in cafeterias and restaurants [138]. Such educational interventions typically highlight the significance of reducing FW and offer practical tips, such as portion control and proper food storage techniques. The initiative was based on the idea that increasing awareness and education about FW issues would lead to less waste. School environments play a vital role in raising awareness and imparting knowledge about food to younger generations. Incorporating FW into specific curricula offers long-term benefits and can be integrated with other food-related subjects [139]. The awareness campaign utilized one-way communication methods, such as posters and table talkers, to inform canteen visitors about the negative aspects of FW and to nudge students to consume food more responsibly. The school conducted educational class lessons for its students, focusing on the ecological consequences of FW, its environmental impact, the scarcity of food resources, and the importance of responsible food consumption. This comprehensive approach aims to raise students’ awareness and positively influence their eating habits [14,120,136].

**Figure 5:**
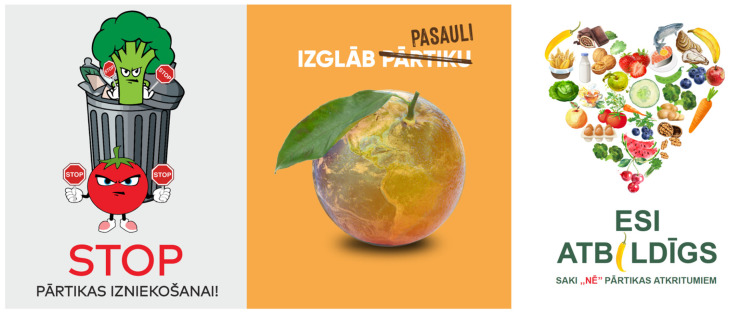
Informative posters were placed in the S2 canteen with slogans like “STOP wasting food!”, “Save the world”, “Be responsible—say no to food waste”.

**Figure 6:**
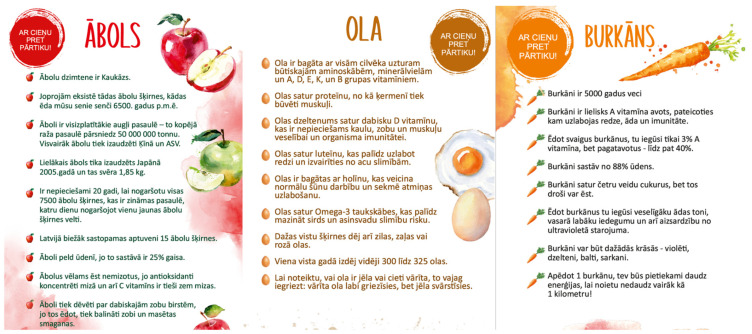
Table talkers were placed in the S2 canteen, featuring nutritional information and interesting historical facts about the origins of food products such as apples, eggs, and carrots.

This process was implemented in the form of class lessons (at least 2 h per academic year in each class from 1st to 7th grade). At the same time, 16 (12 + 4) informative posters were placed in the school canteen, indirectly nudging students toward the responsible consumption of school food. In the school canteen, table talkers were changed every 2 weeks with interesting facts about various school food products (see Figure 5 and Figure 6).

Additionally, to intensify the impact of the awareness and educational campaign, a creative poster competition was organized in the school for 1st- to 9th-grade students on the following topics: “I am what I eat”; “Eat responsibly: think before throwing away”; “I am a healthy eating agent”; and “Spare the planet, do not waste food!”. Participatory nudging interventions for FW reduction encourage students to lead campaigns and create content (e.g., videos, posters) about FW, fostering a peer-driven approach to behavior change. As a result of the competition, four drawings were selected and used to create informative posters, which were then placed in the school canteen.

#### 3.3.3. A Set of Organizational Changes

In S3, the implementation of organizational changes in the provision of catering services included the use of larger diameter plates for serving food, longer lunch breaks, and the presence of a supervising teacher during the lunch break. The chosen interventions were based on the results of our previously implemented research, as it was observed that a school selected used plates of an insufficient diameter, which did not allow students to place the food ingredients in such a way that they did not mix (for example, meat sauce was placed on top of pasta along with vegetables), resulting in spoiling the visual appearance of the food on the plate, which could be one of the factors contributing to PW, especially in primary school. Researcher observations have shown that often the food is mixed during serving, and the schoolchildren refuse it because they do not understand the ingredients of the food. Schoolchildren could refuse to eat or not finish eating the food offered to them if they are not satisfied with the appearance, taste, texture, color, and temperature of the food [123,140,141,142]. Larger diameter plates would allow food to be placed more transparently and be more visually appealing to the schoolchildren, thereby encouraging the acceptance of food by them. The use of larger plates in the intervention relates to school catering in Rezekne City, as the main course is served to each student before the lunch break, and they cannot choose the type and quantity of food.

Regarding the extension of the lunch break, several research studies have concluded that an insufficient lunch break length might be a factor contributing to PW, as a short lunch break does not give the schoolchildren enough time to eat a full meal [136,143,144,145]. Based on the analysis performed within our previous research using artificial intelligence, it was concluded that an optimal lunch break reduces the amount of PW by 20% [106]. Extending the lunch break to at least 30 min and reviewing the school timetable, avoiding the lunch break too early (i.e., until 11:00 a.m.), could contribute to a reduction in PW.

The decision on the presence of the supervising teacher during the lunch break was taken into consideration because the non-involvement of supervisory or support personnel in the catering process (e.g., a teacher or canteen personnel), which could otherwise promote the schoolchildren’s healthy attitudes toward food and new tastes and help to reduce PW, is referred to as one of the factors in PW [123,136,140]. The intervention we proposed is the presence of a class teacher during the lunch break to help and encourage children to eat and try new foods, as well as to stimulate teachers to act as role models, teach the children how to behave in the canteen, and discuss food and nutrition during meals.

### 3.4. Description of the Catering Management and Unified Menu

In all Rezekne City schools, the catering process is organized in closed-type canteens (referred to hereafter as school canteens), supervised by the municipal school board, ensuring compliance with hygiene and healthy nutrition standards, and funded by the local government of Rezekne City. School canteens are equipped to follow safe food handling regulations, and specialized workstations are provided for canteen staff.

In all the school canteens included in the field study, food was partially pre-served on tables designated for each class. Just before the lunch break, canteen staff placed individual portions of the main dish (consisting of staple foods and meats) on plates at the assigned tables. In S4, vegetables with the main dish were served on the same individual plate for each student, while in S1, S2, and S3, vegetables were served in common dishes on the tables for each class separately. In all the schools, the soup was served in common soup pans on the tables for each class, with the amount calculated based on the number of students in each class using standardized measures and serving cups. Slices of bread were placed on tables in common containers according to the number of students in each class. Beverages were served in separate glasses for each student. Fruits (usually whole unpeeled apples, pears, or bananas) were placed on tables in common containers according to the number of students in each class. Similarly, glazed curd cheese was served in its packaging in common containers according to the number of students in each class.

This means that for those students whose catering expenses were covered by the state/municipal budgets, it was not possible to choose the type of food, as the food was already served following all the guidelines regarding the amount of food served, calories, and nutrients. In addition, students could not choose the size of the portion themselves (smaller or larger, depending on their feeling of hunger or age). In all the schools of Rezekne City, catering is provided free of charge for the following students: grades 1–4, for whom free lunches are funded by the national government and grades 5–7, for whom free lunches are funded by the local government of Rezekne City.

Only students in grades 1–7 were included in the field study sample, i.e., those who were entitled to free lunch. During the PW measurement weeks, students in all the schools were fed according to a unified lunch menu, designed for the PW measurement week that would eliminate differences in food availability and ensure laboratory conditions, thereby reducing the influence of external factors on the students’ individual food preferences (see Table 6). The development of the unified menu was based on the results of the previous project, including dishes that students generally liked, disliked, or had a neutral attitude toward. During the PW measurement weeks, the schools ensured that the menus were repeated and the food offered to the students was the same in all the schools. The development process of the unified menu is described in detail in our previous study [27].

### 3.5. Data Collection

Measuring FW is a crucial component of a strategic intervention to reduce FW. It helps to assess the effectiveness of interventions and tracks progress in reducing FW. Additionally, measurement provides consumers with tangible information about the quantity, composition, and cost of the food [38].

In total, PW measurements were taken three times during the school year and performed simultaneously in all four schools, and the students were fed according to a unified menu.

The pre-intervention quantification of PW took place on 25–29 September 2023 in all four school canteens to establish a baseline level of PW. The aforementioned interventions for PW reduction were introduced in three schools after the pre-intervention measurements were taken. On 11–15 December 2023, the first post-intervention quantification of PW was performed to track the effects of the interventions in the short run. It should be noted that PW quantification was also carried out in a control group in S4, where no interventions were implemented. This allowed us to examine whether the test interventions reduced PW or the reductions were due to other trends and factors that would have occurred regardless. The second post-intervention quantification of PW in all four schools was performed on 15–19 April 2024 to track the long-term effects of the interventions.

Each school had a different lunch break schedule. The average lunchtime for grades 1–4 was from 9:30 to 11:30, and for grades 5–7, it was from 11:30 to 13:00. The researchers arrived at the schools at about 9:00 in the morning and finished their work at about 14:00 (depending on the school) for 5 consecutive days of the measurements.

Before the meal, the researchers identified the expected number of students based on the number of main dishes served on the table. During lunch, they registered the actual number of students who participated in the lunch.

The research employed the following methods: observation, photography, and direct manual weighing of PW by food category and by grade of students.

During the PW measurement, the students of the same age group were observed across all schools (grades 1–7), and the amount of PW was identified separately for each class. All the observed schools are located within the same geographical region, specifically in Rezekne City, indicating that the children belonged to the same ethnic group. Pre- and post-intervention PW measurements in all the schools were conducted simultaneously three times over one week (five working days).

During the PW measurements, the students were asked to leave their dirty plates on the tables (usually, the students had to bring them to a special table near the canteen’s dishwashing room). When the students finished their lunch, the researchers gathered the PW into buckets, dividing it into the following categories: soup, staple food, meat/fish, salad/vegetables, bread, fruit, and curd products (glazed curd cheese). The PW measurements were taken separately for each class.

**Figure 7:**
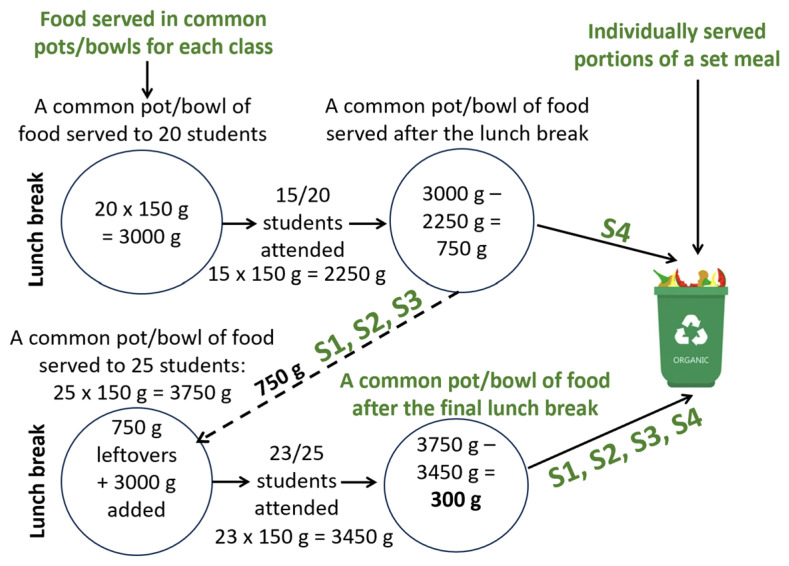
Scheme of the PW generation process in S1, S2, S3, and S4 (an example).

Individually served portions of a main dish (staple food and meats/fish on an individual plate) were not weighed before the lunch break. To calculate the number of main dishes served, the researchers relied on the meal weight indicated in the menu per student (see Table 6). A different approach was applied to measure the amount of soups, salads, and bread served in common bowls and pots for each class separately. In S1, S2, and S3, the researchers recorded the weight of each pot/bowl with soup/salad/bread before the lunch break (gross amount) and the weight of an empty pot/bowl to calculate the net amount of served soup/salad/bread for students. If any soup/salad/bread was left in the common pot/bowl after the lunch break, the pot/bowl was replenished for the next class. The remaining soup/salad/bread in common pots/bowls was discarded only after the final lunch break (see Figure 7). According to the legislation of the Republic of Latvia, school meals are not intended for reheating or reuse the next day. It should be noted that primary school students often took leftover bread to class to consume later.

The situation regarding serving soup in common pots was different in S4. The common soup pots in this school were not replenished for the next classes. Instead, the remaining soup in the pots was discarded immediately at the end of each lunch break, as per the decision of the canteen manager due to hygiene concerns (see Figure 7). The situation was similar with leftover bread in common containers; however, in this school as well, primary school students often took bread with them to class. As mentioned, salad in this school was served to each student individually on main dish plates.

Considering the specifics of catering management in the schools observed, in this study, we define PW as the amount of food served to students that remains uneaten on their individual plates, as well as discarded food leftovers in common bowls and pots. The total measured weight of PW includes uneaten food left on individual plates and discarded food in common bowls and pots following the free lunches provided to students in grades 1–7 in the Rezekne City schools observed.

After each lunch break, all buckets with the PW were weighed, and the data were entered into a waste registration protocol. The following measurement tools were used to quantify the PW: two kinds of high-density polymer buckets (a large bucket with a capacity of 2 L, weight 61 g, and a small bucket with a capacity of 1 L, weight 35 g; each bucket was marked with the food category and the number of the class for which it was intended and electronic kitchen scales were used (model—Clatronic KW3412, art. No. 271680, measuring range—up to 5 kg, units of measurement—grams, producer Clatronic International GmbH, Kempen, Germany).

## 4. Data Analysis and Results

All of the data analyses were performed using the statistical software R and MS Excel. The research employed a statistical analysis method, the Wilcoxon signed-rank test, using the R method “wilcox.test” to test the null hypothesis and verify sufficient differences between two paired groups, namely pre-intervention and post-intervention groups.

**Figure 8:**
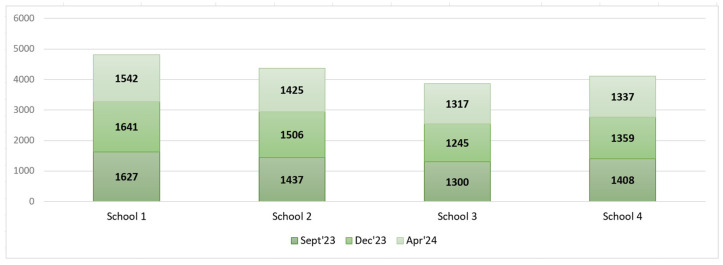
Distribution of the number of samples by school at each PW measurement (i.e., the actual number of students who participated in lunch breaks).

In total, 17,144 plates (number of samples) were surveyed in three PW measurements, with 5772 in September 2023, 5751 in December 2023, and 5621 in April 2024. The distribution of the number of surveyed samples by school is shown in Figure 8.

### 4.1. Data on Total Food Served and PW

**Figure 9:**
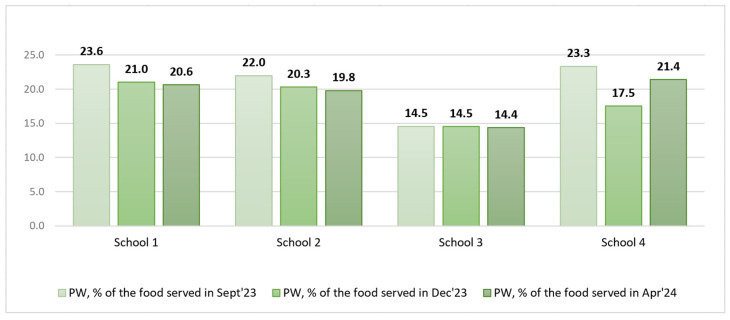
Total PW as a % of the food served.

Initially, the total amount of PW was calculated as a ratio of the food served, expressed as a percentage, including soup, salad, and bread served in common pots/bowls (see Table 7 and Figure 9).

**Figure 10:**
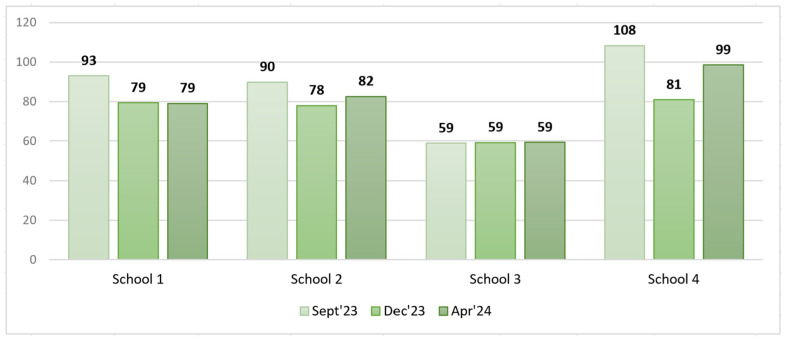
Total PW, g/student (based on the actual number of students who participated in lunch breaks).

An analysis of pre- and post-intervention total PW as a % of the food served did not allow us to draw conclusions on the impact of the interventions, as the total amount of food served in the communal dishes varied significantly from week to week, especially that of soup. It was therefore important to express PW in grams per student so that the values were comparable (see Figure 10).

An analysis of the obtained PW results in grams per student revealed that the highest PW was registered in S4. This could be justified by the fact that the way of serving soup and salads in S1, S2, and S3 differed from that in S4 (see Figure 7), i.e., in S1, S2, and S3, the common soup pot/salad bowl was replenished after each lunch break for the next classes, while in S4, the remaining soup in the pots was discarded immediately at the end of each lunch break and the common soup pots were filled again for the next classes; additionally, in S4, salad was served on the individual plates to each student.

However, a more detailed analysis of the data on the amount of food served—particularly soup in common pots—revealed that the quantity served in some schools (notably S1 and S2) often differed significantly from the amount specified in the menu per student. In some cases, the difference between classes during the same lunch break was as much as double. This imprecision in food serving can significantly affect the relative amounts of PW (as a % of the food served). Conversely, in S4, no measurements were made of the soup served in common pots, as in this school, the serving waste was discarded after each lunch break.

### 4.2. Data Filtering

To eliminate the impact of different approaches to serving food in common pots/bowls, data analysis was conducted using data filtering. This involved excluding soup, salad, and bread waste, as well as intact discarded portions from the statistical analysis and including only PW data on the main dish (staple food with meat/fish), fruits, pastry items, and glazed curd cheese.

It is also important to note that in all the schools, some portions of food served remained intact on the tables, and some of the intact portions were eventually discarded. This was mainly owing to inaccurately planning the expected number of students, as information from parents about their children’s absences did not always reach the canteen administration in time. As a result, surplus portions were prepared and served based on an incorrect estimate of the expected number of students. Some children might not have liked the food and might not have touched their portions. The surplus portions were sometimes partially eaten by classmates, sold to senior students, or discarded, especially toward the end of the day. We excluded such intact discarded portions from both the amount of food served and the amount of PW to ensure the accuracy of the data analyzed. The PW data obtained were then expressed in grams per student by dividing the total amount of PW by the actual number of students who participated in lunch breaks (or the number of samples).

By filtering the PW data, we focus on the more consistent and comparable elements of the meal, providing a clearer picture of waste patterns. Measuring soups can be complicated due to their varying properties, such as significant differences in viscosity, ingredient composition, and portion sizes, which make accurate comparisons across different meals and schools challenging. It should also be taken into account that soup consumption could be highly variable among students due to personal preferences [146,147,148].

**Figure 11:**
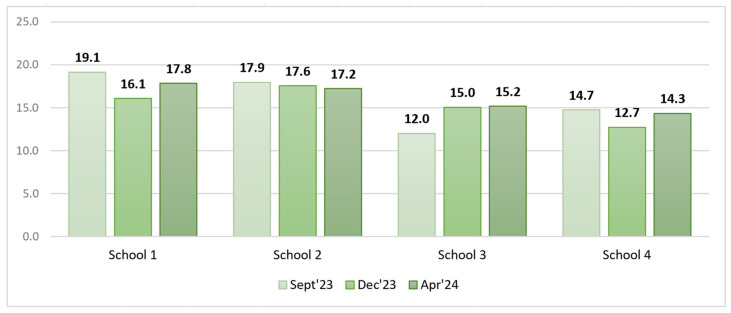
Filtered PW data as a % of the food served.

**Figure 12:**
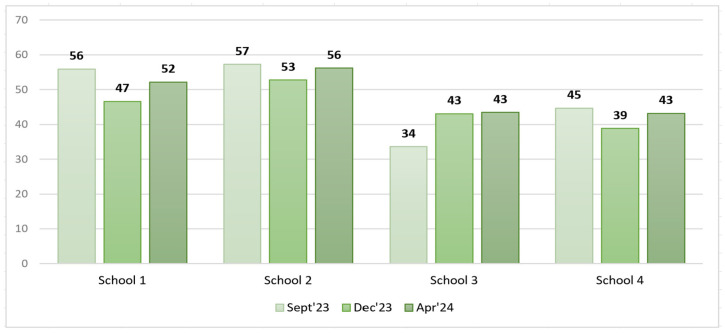
Filtered PW data, g/student.

The filtered data on food served and PW after excluding soup, salad, bread, and intact and thrown-away portions from the analysis are available in Table 8 and Figure 11 and Figure 12.

An analysis of the filtered PW data in grams per student revealed that in S1, S2, and S3, the PW decreased in the short run, whereas in the long run, a slight increase was observed. In contrast, in S3, we could observe a PW increase in the short run that remained unchanged in the long run.

Part of the reason for the long-run result was that in S2 and S4, a significant problem arose with catering management related to serving the exact number of portions according to the expected number of students who were going to participate in the lunch break on a given day. It should be noted that no digital tools were used in any school to collect information about the expected number of students and pass it to the canteen. Usually, the key persons receiving and passing the information were the class teachers who were informed about the schoolchildren’s absences by the parents, and then the class teacher either directly informed the canteen personnel early in the morning or the school nurse collected this information and then passed it to the canteen personnel.

If the information about the expected number of students is incorrect, then surplus portions that exceed the actual number of students are served on the tables. Usually, surplus portions are (partially) eaten by classmates, most often eating only the meat dish, and leaving the side dish on the plate, which goes to waste. If such surplus portions are intact, they can be given to senior students or sold to students/school personnel. Some of the surplus portions also end up in the garbage. Considering the number of surplus portions recorded in S2 and S4 (see Table 9), it obviously affected the PW fluctuations.

### 4.3. Class View and Day View Analysis Models

To draw unambiguous conclusions on the results of interventions, the filtering of PW data allowed us to apply two analysis models, namely Model 1 (class view), to analyze the pre- and post-intervention filtered PW data per student for each school and each class over a total of five days and Model 2 (day view), to analyze the pre- and post-intervention filtered PW data per student per day for each school for all classes combined.

Model 1 (class view) allows us to analyze the pre- and post-intervention filtered PW data, g/student, for each class in the short run (September 2023–December 2023) and in the long run (September 2023–April 2024). For each class, the filtered PW data, g/student, make a statistical pair, with September 2023–December 2023 and September 2023–April 2024.

There were 15 pairs for S1, 17 for S2, 16 for S3, and 15 for S4. To identify the impact of the interventions, the null hypothesis “pre- and post-intervention filtered PW data, g/student, remain unchanged” was tested. The rows represent classes (1a, 1b, 2a, etc.), while the columns represent filtered PW g/student datasets (September 2023–December 2023 and September 2023–April 2024) (see Table 10 and Table 11). The hypothesis was tested individually for each school. A Wilcoxon signed-rank test with a confidence level of 95% was performed.

**Figure 13:**
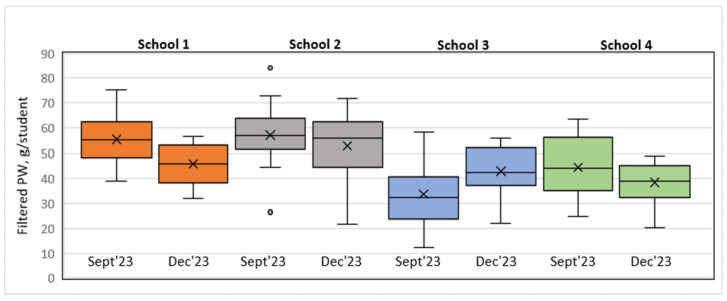
Analysis of filtered PW data in the short run, g/student, September 2023–December 2023 (class view).

Model 1 (class view) results: In the short run for S1, a Wilcoxon signed-rank test with a *p*-value = 0.000 < 0.001 indicated that there was a statistically significant difference in the filtered PW data, g/student, between September 2023 and December 2023, thereby indicating a significant change in PW in the case of the plate waste tracker intervention. Figure 13 shows that the plate waste tracker intervention reduced PW in the short run. Statistically significant differences in PW were also found for S3 (*p*-value = 0.011 < 0.05), where several organizational changes in the catering process were introduced; yet, in this school, the opposite was observed, i.e., an increase in PW (filtered data, g/student) in the short run (see Figure 13). In the cases of S2 (*p*-value = 0.159) and S4 (*p*-value = 0.095), there was not enough evidence to reject the null hypothesis, and it was assumed that for these schools, the permanent difference in PW was not statistically significant in the short run.

**Figure 14:**
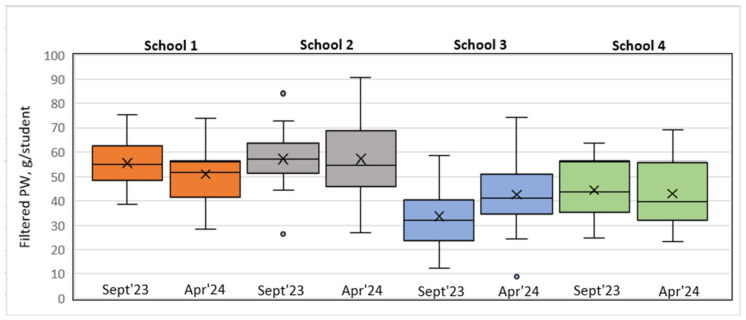
Analysis of filtered PW data in the long run, g/student, September 2023–April 2024 (class view).

In the long run (September 2023–April 2024), however, the Wilcoxon signed-rank test showed that in the cases of S1 (*p*-value = 0.107 > 0.05), S2 (*p*-value = 0.890 > 0.05), and S4 (*p*-value = 0.639 > 0.05), there was no statistically significant difference in PW (filtered data, g/student), which means that the plate waste tracker intervention in S1 and the awareness and educational campaign intervention in S2 did not have a significant effect. S4 was the control, with no interventions implemented, thereby having no significant effect of external factors on the result (e.g., seasonality). In contrast, in the case of S3, a significant difference in PW (filtered data, g/student) (*p*-value = 0.004 < 0.01) was found, with the PW increasing (see Figure 14). The results for S2, S3, and S4 were consistent with the short-run results, while in the case of S1, the result changed. The intervention in S1 showed a decrease in PW (filtered data) in the short run but not in the long run, as the result was statistically insignificant (*p*-value = 0.107 > 0.05).

Model 2 (day view) analyzed the pre- and post-intervention filtered PW data per day for each school for all classes combined. In this case, the rows represent the days of the week (Monday–Friday) and the average PW, g/student, at all the schools. For each school, there were five data pairs in the short run (September 2023–December 2023) (see Table 12) and in the long run (September 2023–April 2024) (see Table 13). The null hypothesis was the same: “pre- and post-intervention filtered PW data per student remain unchanged”. A Wilcoxon signed-rank test with a confidence level of 95% was performed.

**Figure 15:**
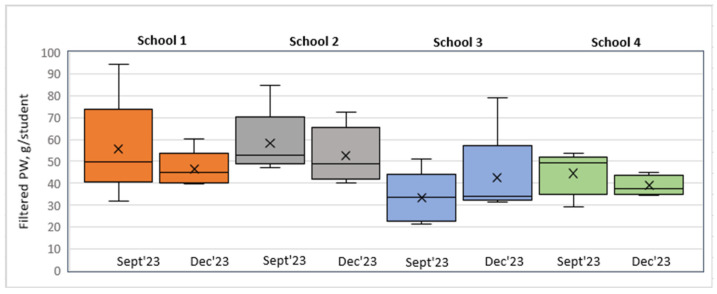
Analysis of filtered PW data in the short run, g/student, September 2023–December 2023 (day view).

**Figure 16:**
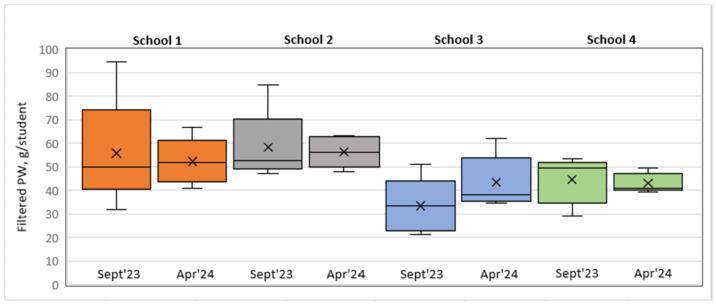
Analysis of filtered PW data in the long run, g/student, September 2023–April 2024 (day view).

Model 2 (day view) results: In the short run, as well as in the long run, the Wilcoxon signed-rank test indicated that there were no statistically significant differences in PW (filtered data, g/student) between all the schools, meaning that the interventions implemented did not have a significant effect on changes in PW. However, it is important to note that in the case of S3 in the long run, the *p*-value = 0.063, which was close to the threshold of 0.05, meaning that there was probably some PW difference, but the evidence was not strong enough. As shown in the boxplot short run diagram (see Figure 15), the post-intervention case in S3 indicates a tendency toward increasing PW. A similar situation is seen in the long-run diagram (see Figure 16); however, the S3 long-run *p*-value = 0.063 is more significant than the short-run *p*-value = 0.188.

## 5. Discussion

In the short run (September 2023–December 2023), Model 1 (class view) revealed that the PW reduction intervention was effective in S1, where a plate waste tracker was installed, as the amount of PW (consisting of the main dish (staple food with meat/fish), fruits, pastry items, and glazed curd cheese) (filtered data, g/student) significantly decreased. In the case of S3, a significant difference in PW (filtered data, g/student) was also found; however, it cannot be concluded that the intervention had a positive effect because the PW, g/student, increased. In this case, the impact of external factors such as competitive food cannot be excluded because with the extension of the lunch break, students who do not like free lunches have enough time to buy and eat other food in the school canteen that is available for money outside the free lunch menu, meaning that in this case, the free lunches served are more likely to be thrown away. No in-depth analysis of the S3 situation was performed to unequivocally conclude the factors in the increase in PW (filtered data). It should be noted that the earlier 20 min lunch break was restored in S3 after the end of the field study in the study year 2024/2025. The statistical analysis showed no statistically significant change in PW (filtered data, g/student) after the awareness and education campaign intervention in S2. S4 was the control, and no effect of external factors was observed there.

In the long run (September 2023–April 2024), the statistical analysis did not show statistically significant changes in PW (filtered data, g/student) after the interventions in S1 and S2. The exception was S3, where according to Model 1, the opposite effect was observed, i.e., an increase in PW (filtered data, g/student). S4 was the control, indicating the absence of relevant external factors that could have influenced the experimental results.

Model 2 (day view) showed no statistically significant differences in the amount of PW (filtered data, g/student) for all the schools in the short run and the long run (see Table 12 and Table 13).

After summarizing the results provided by Model 1 and Model 2, it should be noted that a significant difference between Model 1 and Model 2 was the number of statistical pairs to be analyzed, which tended to affect the accuracy of the analysis results (the higher the number of pairs, the higher the accuracy). In the case of Model 1 for S3 in the long run, the *p*-value = 0.004 was more accurate, and if corrected for accuracy under Model 2, the *p*-value = 0.063 for S3, which was close to 0.05, suggesting that there was still a statistically significant difference between the pre- and post-intervention PW (filtered data, g/student) in the long run. In the case of S1, a similar correction is doubtful, since in the short run for S1, the *p*-value = 0.000 under Model 1 and the *p*-value = 0.313 under Model 2.

What is the semantic difference between Model 1 and Model 2? Under Model 1 (class view), a particular class was the subject of observation, which was therefore more precise in terms of both data and methodology. In contrast, Model 2 (day view) considered the statistically average student deciding to eat or not to eat the school food served according to the free lunch menu. Analyzing the responses of 13,584 students (which, according to approximate calculations, account for 30% of the total number of students who participated in lunch breaks during the intervention period) provided through the plate waste tracker regarding the reasons for PW, the most frequently mentioned reason was “I am full” (44.3%), followed by “I did not like it” (38.8%), “I did not have enough time to eat” (9.6%), and “The portion size was too large” (7.3%). Combining the responses “I am full” and “The portion size was too large”; it is evident that the primary reason for PW (in 51.6% of cases) is directly related to the quantity of food served. The second significant reason for PW is students’ preferences and dislike of the menu (38.8%). Given that Model 2 dealt with a menu that changed daily, it can be concluded that in the case of S1 in the short run, there was a high probability of being affected by factors arising from students’ food preferences. Most likely, the short-term reduction in PW was driven by the installation of the plate waste tracker and its associated psychological effects on students and their desire to reduce PW. However, in the long run, the inability to choose the size and type of food supported the hypothesis about the absence of a sustained impact of the plate waste tracker intervention.

In the case of S3 under Model 2, if corrected for accuracy and assuming that there was still a change in the amount of PW (filtered data, g/student) in the long run, it could be concluded that there was a probability of an effect of the student food preference factor on the result. It could be assumed that during the S3 intervention, with the longer lunch break of 30 min, if a student did not like the free lunch, they had enough time to purchase other foods outside the free lunch menu, which might explain the S3 anomaly with higher amounts of PW (filtered data, g/student) in the short run as well as in the long run. For example, a previous research study found that 41.6% of the students decided to reject food if they did not like it. However, competitive food in schools affects students’ satiation in 21–42% of cases, and they eat at best 1/3 of the portion served [106]. The effect of external factors such as food seasonality was unlikely, as no significant difference was observed for S4; therefore, it was more likely that the increase in PW (filtered data, g/student) in S3 was due to an in-school factor.

The results obtained in the study should be interpreted through the prism of the MOA framework (Figure 2) to better understand the prerequisites of students’ FW behavior. In the out-of-home catering model, FW is primarily determined by activities related to ordering/serving and consuming. In school catering, students’ impact is limited to these stages, where factors such as portion size differentiation, the choice of a food type, and eating behavior significantly influence the amount of FW produced. It is important to provide students with opportunities to consume school meals responsibly, which involves tailoring portion sizes to their needs based on appetite level and physiology, food choice options, and a takeaway option for uneaten food. By projecting this model onto the catering organization in Rezekne City schools, we can conclude that in this case, the model lacks the Opportunities element, because first- to seventh-grade students are served free lunches according to the same menu without the option of choosing the type of food, without differentiating the size of the portions depending on their age and appetite, as well as without providing the possibility to take away uneaten food. Even though the student is motivated to consume food responsibly, and they can do it by having appropriate knowledge and skills (in-home circumstances), they do not have opportunities to act responsibly with food in the school canteen.

This conclusion also represents the result observed in the schools surveyed. The interventions implemented in S1 and S2 could not produce a full effect, as the catering model in Rezekne City schools was not adaptable to students’ food preferences, age, appetite, physiology, etc. However, many authors point out that it is important to take into account children’s food preferences through the implementation of new menus that have been designed based on the results of student food satisfaction/food preference questionnaire surveys [149]. PW in school canteens is influenced by students’ menu preferences, shaped by individual and contextual factors [140]; therefore, more proactive menu management by developing more appealing menus can be an effective strategy to boost food consumption and reduce PW [99,150,151]. For instance, the present research found that the amount of waste consistently spiked on Thursday, with the average amount of PW (filtered data, g/student) being 37% higher than the average for all three measurements. This increase was largely due to a side dish “stewed rice with carrots and corn” that was not preferred by the students because of the vegetables added to the rice. A FW analysis by component can help to identify foods with the highest PW, allowing for their improvement or modification in menus [89,151,152].

Several studies on the reasons for PW in schools with a similar pre-served meals catering model confirm that the amount and type of food served are among the main contributors to PW. For instance, Sehnem et al. [153], analyzing FW in seven schools in Brazil with a pre-served set meals catering model, found that approximately one-fifth (20%) of the food remained uneaten on plates. Boschini et al. [119], in their analysis of 78 primary schools across three regions in Italy with pre-served set meals, found that PW increased with larger portion sizes. They identified a threshold of 370 g/day per capita for served portions, above which PW grew significantly. Also, Favuzzi et al. [154] found that the weight of the food served influences FW. It should be noted that in the study by Favuzzi, as in our study, all children were served a standard portion size using a standardized graduated ladle [154].

If catering is organized in the form of pre-serving or pre-portioning (as was the case mainly in the schools surveyed), it is important to adapt the amount of food served to the physiology of students; if it is not possible to serve food according to their appetite level, at least their age needs to be considered. Currently, any school menu is designed for students of all grades entitled to free lunch and the weight of the food is the same for all, regardless of age. The present research did not analyze differences in PW, g/student, between students of different ages; however, even without any further statistical analysis, a difference in PW (filtered data, g/student) between primary and upper secondary school students could be identified. The latter had a lower average amount of PW, g/student, which we plan to analyze in the future. Some researchers note that the sex of the child also tends to influence the amount of FW, e.g., Favuzzi et al. [154] found that meal judgment is not the only factor contributing to FW, identifying larger amounts of FW, particularly among females in primary school, even when they expressed a positive opinion about the meal, and they concluded that the increase in FW could be attributed to the surplus portions served to female students.

Steen et al. [155] found a positive correlation between the amount of FW (both plate and serving waste) and the portion size regardless of gender, especially when older students take more food on their plates than they can eat. Often, this is the case of food overproduction (and therefore also overserving) due to the lack of information about the daily number of diners [155]. Our research observed that in S2 and S4, the expected number of students was often larger than the actual one; therefore, surplus portions were served, and some of them were discarded, leading to higher PW amounts.

Referring to the implementation of FW interventions in schools, it should be noted that in our case, the interventions did not work for several reasons. First, two schools tried single interventions, namely the plate waste tracker in S1 and an awareness and educational campaign in S2.

Malefors et al. [74,114] found that the plate waste tracker intervention in Swedish schools was effective in significantly reducing both PW and serving waste. This tool provided real-time feedback to students on the amount of food they wasted, which not only decreased PW by 37% but also led to a substantial 62% reduction in serving waste as a spillover effect, demonstrating its impact on overall waste reduction in school canteens [74,114]. The installation of the plate waste tracker in 12 schools in Sweden and Germany, featuring a buffet serving style, effectively reduced PW by 17%, significantly lowering environmental impacts and nutrient losses while demonstrating long-term sustainability and cost-efficiency [156]. Undeniably, the research studies by Swedish colleagues clearly revealed the plate waste tracker as a disruption in daily routine [38] and the effect of nudging on the food consumption behavior of students, as the students had such an opportunity because it was self-service catering (buffet meals) in the schools observed. In our research, the effectiveness of the plate waste tracker was short-lived, largely due to the unadaptable catering organization model in S1. This lack of flexibility significantly limits the potential of the plate waste tracker to influence student behavior in the long term. While the initial introduction of the tool may have created a psychological impact, encouraging students to reduce PW temporarily, the inability to align portions with individual needs ultimately undermined its sustained effectiveness. This limitation highlights the importance of integrating adaptable catering practices, such as allowing portion customization or offering self-service options, to fully leverage the benefits of interventions like the plate waste tracker. If this obstacle is overcome, further actions to enhance the tracker’s effectiveness include installing multiple devices in canteens to avoid bottlenecks, reducing queues, and ensuring younger children can interact with the tracker without feeling rushed. Simplifying the interface with child-friendly visuals can make feedback more engaging and accessible. Gamified elements, such as class competitions rewarding waste reduction, could further motivate participation. Regular monitoring and feedback loops are essential, while integrating tracker insights into lessons on sustainability and healthy eating can deepen students’ understanding and foster mindful food consumption.

Favuzzi et al. [154] did not identify a strong impact of educational intervention on the amount of waste generated in school canteens, indicating that a single educational effort, regardless of its complexity, is insufficient to produce significant changes because after just one educational intervention, both parents and children tend to revert to their habits afterward, which might explain the slight and insignificant difference in waste observed before and after the intervention.

A comparison of two interventions to reduce PW in university canteens by Visschers et al. [137] revealed that providing information about FW alone did not lead to any reduction in waste. However, when smaller servings were offered alongside the informational campaign, PW was reduced by 20%. This suggests that portion control, combined with awareness efforts, is more effective in minimizing FW.

In turn, Liz Martins et al. [157], analyzing PW changes after a 6 h children’s nutrition education intervention in three primary schools in Portugal with a pre-served catering model, observed a significant reduction in PW in the short term (one week after the intervention), particularly for soups and main dishes. However, the effect diminished in the medium term (three months after the intervention), highlighting the need for ongoing reinforcement.

In our case, due to the specifics of the research project, it was not possible to implement an intensive educational campaign in S2; therefore, only two lessons were delivered per school year in each class from first to seventh grade, which was insufficient. For instance, in Italy, three classes spent four hours per week for five weeks on a comprehensive awareness program, creating posters on FW, and exploring related topics such as climate change and biodiversity through follow-up activities [158]. In Bari, for the educational intervention targeting children, a flipped classroom method was employed during one month. In total, 361 students in 12 schools first engaged in autonomous learning at home, followed by applying their new knowledge in the classroom under teacher guidance [154]. In our study, the allocation of only two hours of teaching per school year represents a significant research limitation. Such limited exposure was likely insufficient to foster sustained behavioral change, as highlighted by the existing literature that underscores the necessity of comprehensive and continuous educational efforts to effectively influence food waste reduction behaviors. The short duration of the intervention may have made it harder for students to absorb the key messages, which is important for building long-term habits. This limitation likely contributed to the lack of statistically significant reductions in PW in S2, emphasizing the need for more intensive and recurrent educational initiatives to achieve meaningful and lasting outcomes in future interventions.

However, 16 posters with slogans for responsible food consumption and reducing FW were displayed in the S2 canteen as a nudging intervention. Whitehair et al. [159], in their study of 19,046 trays in a university dining operation, concluded that a simple to-the-point prompt-type message reduced FW by 15%. It should be noted that unlike students in our research, university students were able to adjust the amount of food they put on their plate, so they could change their food consumption behavior influenced by nudging. In our research, the students did not have this possibility; therefore, we expected that they would simply start eating better under nudging, but this did not happen because it was impossible to force a child to eat all the food offered if they did not like it, had no appetite, or the portion was too large. Another nuance that should be noted is FW messaging on posters. Nisa et al. [160] assessed FW messaging for households and found that more forceful messages (e.g., “stop waste” or “don’t waste”) on posters might be ineffective and potentially counterproductive, as they were more likely to trigger psychological resistance compared to softer persuasive messages like “reduce waste”, which were perceived as less controlling and authoritative. Of the 16 posters displayed in S2, six had the following slogans: “Be responsible—say no to food waste”; “Use food responsibly! Don’t throw it in the garbage!”; “School food is healthy and tasty. Say no to food waste”; “Don’t waste, respect food, respect nature, save money”; “STOP wasting food”; and “Respect food. Say no to food waste!”. We assume that in this case, there might be a trigger effect on the students.

The organizational changes implemented in S3, including the use of larger plates, extended lunch breaks, and the presence of a supervising teacher during meals, did not yield the expected results; on the contrary, filtered PW g/student amount increased. We are inclined to associate the anomaly of PW increase with the S3 lunch break extension to 30 min, which may have allowed students to buy other food outside the school’s free lunch menu, which caused the free lunches served to end up in the garbage.

Despite the fact that one of the widely used FW reduction interventions in out-of-home catering is smaller plates so that food consumers can put less food on their plates [33,161], it should be noted that it is useful under the self-service catering organizational model. However, in Rezekne City schools, including in S3, students are given pre-served main dishes; therefore, using larger plates is beneficial, as it allows students to see clearly and understand the ingredients of the food being served (more engaging for younger students) [27,108,109].

It was difficult for researchers to assess the impact of supervising teachers during mealtimes in S3. The SKOOL report emphasizes that those who supervise students during meals are key to reducing waste, making it essential to provide them with the necessary skills. While it might seem simple, motivating all personnel to participate is challenging. Supervisors need the knowledge and resources to guide students in reducing waste while understanding their preferences and encouraging them to try new foods [158]. School principals, canteen supervisors, and teachers play a crucial role in facilitating, designing, and implementing waste minimization interventions, with the human factor emerging as the most significant element in reducing FW. The lower FW amounts were observed in areas where students had greater awareness, driven by two key factors, namely the integration of sustainable eating behaviors into their routines and the strong focus on sustainability by school managers and teachers [14]. In Liz Martines’ study [157], an intervention focused on educating teachers about FW and encouraging their active presence during lunch, implemented in a Portuguese school with a pre-served catering model, demonstrated a better impact in the medium term. It led to a slight but consistent reduction in PW over time, indicating that teacher-focused interventions had more sustained effects in the medium term. During the three months following the start of the intervention, teachers were encouraged to be present during lunchtime as much as possible and to promote waste reduction among students actively.

In our study, the supervising teachers during lunch were class teachers who had not received any prior training on the issue of FW. This could also be regarded as a limitation of our research, which may have limited the effectiveness of the intervention, as the class teachers were not equipped with strategies to reduce FW or encourage sustainable eating habits. Without proper guidance, they may have missed opportunities to influence student behavior, such as promoting the acceptance of new foods or reducing waste. This highlights the need for targeted training and clear protocols for supervisors in future interventions to ensure consistency and maximize impact.

The International Food Waste Coalition report “School Kitchen Organization Optimization Learning (SKOOL report)” has admitted that collaborative efforts are more effective in reducing FW than single ones. For instance, educating students about FW and teaching them simple ways to reduce it in the canteen will yield limited results if meal organization, portion sizes, and recipes remain unchanged [158].

Complex FW-reducing interventions are often seen as more effective than single ones, as FW is influenced by various factors. However, evidence is mixed, with some studies showing positive results from combined messages but lacking clarity on which specific element was effective [33]. To drive significant FW change, a combination of targeted interventions, informed by models such as the MOA framework, should be employed to address specific consumers’ behaviors [35].

## 6. Conclusions

The interventions tested in the present research provide valuable insights into strategies for reducing PW in school canteens, particularly within the Latvian context. The findings demonstrate that specific targeted actions can lead to meaningful reductions in FW, though the effectiveness of the interventions can vary depending on the type of intervention and specific conditions under which they are implemented.

The plate waste tracker intervention in S1 resulted in a statistically significant reduction in PW in the short run, highlighting the potential of technology-driven solutions as a means of nudging students’ food consumption behavior through disruptions in their daily routines. The short-term effect can be attributed to the initial curiosity sparked by the installation of the device in the school canteen, which motivated students to try not to leave uneaten food on their plates. However, the long-term data indicate that this reduction is not sustained, suggesting that while such tools can create immediate impacts, their effectiveness might diminish over time without continuous reinforcement or additional complementary measures.

In S2, the awareness and educational campaign showed mixed results. While this intervention is crucial for fostering long-term behavioral change and raising awareness about the importance of reducing FW, the lack of a statistically significant reduction in PW in both the short and long run suggests that awareness educational efforts alone might not be enough. This finding aligns with previous studies indicating that awareness-raising activities need to be part of a broader, more intensive, and complex approach to be effective.

The intervention implemented in S3, stemming from the specific catering organization model used in Rezekne City schools, included organizational changes such as using larger plates, extending lunch breaks, and involving supervising teachers. However, these changes did not yield the expected reductions in PW. On the contrary, an increase in PW was observed in both the short and long run, and the significance of this difference increased over time, as noted in the cases of both Model 1 and Model 2. This outcome underscores the complexity of FW behaviors and suggests that while changes to the dining environment and schedule are important, they must be carefully designed and monitored to avoid unintended consequences.

We cannot conclusively state that the increase in PW in S3 was caused by the implemented intervention, as this is an internal factor, and the experiment would need to be replicated in other schools to generalize the effect to other schools in Latvia that have a similar free school meal catering model. A similar situation applies to the plate waste tracker intervention in S1, which had a positive impact in the short run, but the experiment should be replicated in several other schools with similar catering models before widely implementing this device.

Reducing PW in Rezekne City schools requires a combination of targeted interventions and structural adjustments. Flexible approaches to portion sizes are essential, as rigid, pre-determined servings often result in increased waste. Customizable portioning practices that account for students’ age and appetite can help address this issue. Transitioning to self-service or semi-self-service catering models could reduce the mismatch between servings and consumption by giving students greater autonomy in portion selection. Digital tools for meal planning could enhance efficiency by allowing students or parents to pre-select meals, helping canteens better anticipate demand and minimize surplus food. Comprehensive educational campaigns—such as interactive workshops, farm-to-table programs, and competitions—can foster sustainability awareness and encourage responsible consumption among students. The active involvement of teachers and staff is crucial. Training programs can equip them with strategies to promote sustainable eating habits and model responsible behavior during meals. Enhanced monitoring and feedback systems can track trends in PW, guide menu adjustments, and reinforce waste reduction efforts through regular communication with stakeholders.

Overall, the research has confirmed that reducing FW in schools is a multifaceted challenge that requires a combination of interventions tailored to specific contexts. The variability in results across the schools suggests that a one-size-fits-all approach is unlikely to be effective. The research also highlights the need for further studies to explore the long-term sustainability of the interventions and their adaptability to different cultural and operational contexts. By continuing to refine and test the approaches, stakeholders can develop more effective strategies for reducing FW in schools, thereby contributing to broader efforts to promote sustainable food consumption and achieve the global sustainability goals.

## Figures and Tables

**Table 1:** FW in the EU and Latvia by sector of activities, 2021 (based on [8]).

Sectors of Activities		Total FW	Primary Production	Processing and Manufacturing	Retail and Other Distribution of Food	Restaurants and Food Services	Households
EU ^1^	tons	58,400,000	5,100,000	12,400,000	4,200,000	5,400,000	31,000,000
	%	100	8.7	21.2	7.2	9.2	53.1
	kg per capita	131	11.4	27.8	9.4	12.1	69.6
Latvia ^2^	tons	245,442	30,592	32,518	16,765	28,617	136,950
	%	100	12.5	13.2	6.8	11.7	55.6
	kg per capita	130	16.3	17.2	8.8	15.2	72.3

^1^ 2020 data presented; ^2^ definition differs for some figures.

**Table 2:** Interventions to reduce consumer FW (based on [36]).

Category	Policy Option	Description
Information	Information and awareness-raising campaigns	Public campaigns to educate consumers about the impacts of FW and encourage behavior changes
	Social norm campaigns	Campaigns that leverage social norms to influence consumer behavior by showcasing what others are practicing to reduce FW
	Education/skill training	Programs to enhance consumers’ skills in meal planning, food storage, and creative cooking to reduce FW. This includes school programs and public workshops
	Prompts	Visual or verbal reminders placed in strategic locations (e.g., refrigerators, shopping lists) to encourage behaviors that reduce FW
	Feedback	Providing consumers with information about the amount of food they waste, potentially through apps or smart bins, to increase awareness and drive behavior change
	Commitment	Encouraging consumers to make public pledges or commitments to reduce FW, enhancing accountability and consistency in behavior
	Apps and ICT tools	Digital tools that provide information, tips, and incentives to reduce FW, such as apps offering recipes for leftovers or tracking food inventory
Regulation	Regulation on date marking	Standardizing and clarifying date labels (e.g., “use by” vs. “best before”) to reduce consumer confusion and unnecessary waste
	Promotions, product presentation, and packaging	Regulating promotional activities (e.g., banning “Buy One Get One Free” offers) and requiring appropriate portion sizes and packaging that reduce overbuying and waste
	Influencing consumer behavior through regulation targeted at other actors	Adopting regulations that indirectly affect consumers, such as relaxing marketing standards for cosmetically imperfect produce, increasing the availability of surplus food products, and prohibiting supermarkets from discarding edible food
Economic Instruments	Fees and taxes	Implementing pay-as-you-throw schemes that charge households based on the amount of waste they produce, incentivizing FW reduction
	Subsidies	Providing financial incentives for activities that reduce FW, such as subsidies for food donation programs
	Penalties for supermarkets wasting food	Imposing fines on supermarkets that discard edible food, encouraging better food management practices
	Financial incentives for donating food	Offering tax breaks or other financial benefits to businesses that donate surplus food
Nudging and Choice Architecture	Altering food placement in stores and dining facilities	Strategically placing food items in stores (e.g., at eye level) and adjusting serving sizes in dining facilities to encourage the purchase and consumption of appropriate amounts
	Changing serving sizes and portion control	Introducing smaller portion sizes by restaurants and canteens to reduce the likelihood of food leftovers and waste
Voluntary Agreements	Public–private partnerships	Collaborative efforts between the government and private sector stakeholders to implement FW reduction practices
	Industry-led initiatives	Voluntary commitments by businesses to adopt practices that reduce FW, such as improved inventory management and offering surplus food for sale
	Non-binding guidelines and strategies	Developing and promoting best practice guidelines for reducing FW, which businesses and organizations can choose to adopt

**Table 3:** Types, subtypes, descriptions, and examples of the consumer FW prevention interventions (based on [38]).

Type	Subtype	Description	Examples
Nudges	Tools and prompts for food storage and preparation	Interventions, including digital tools, physical aids, and informational campaigns, designed to help individuals manage food more effectively, reduce FW, and promote sustainable consumption	Food trainer app test (United Kingdom) [39]
			Use It Up Tape—a visual prompt for leftover consumption (Australia) [40]
	Other nudges for household FW	Interventions that use strategies like social influence, feedback, awareness campaigns, and innovative tools to raise awareness and encourage behavior change, aiming to reduce FW at the household level across various stages	FW calculator (Finland) [41]
			Study on eco-feedback device (Canada) [42]
	Labeling and visual cues on food packaging	Interventions aimed at reducing FW by improving consumer understanding of expiration dates and promoting better food handling through stickers, time temperature indicators, and storage advice	Day-on-date label (United Kingdom) [43]
			Evaluation of date labeling campaign encouraging consumers to look–smell–taste (Canada) [44]
	Nudges out of the home	Interventions targeting FW reduction and sustainable behaviors in public spaces like schools, restaurants, and hotels; primarily target serving and consumption stages, aiming to influence behaviors like portion control and food storage	Nudging strategies in school canteens (Spain) [45]
			Posters displaying social norms (France) [46]
Education and training	School programs	Interventions engaging students, teachers, and parents in activities like food preparation, creative projects, and using teaching materials to reduce FW and promote sustainable practices in schools and households	Food and nutrition education program (Netherlands) [47]
			Green Chef—a youth-targeted competition (Portugal) [48]
	Training for food business personnel	Interventions educating food industry employees to reduce FW, improve food management practices, and promote sustainability through tailored strategies like menu design, storage optimization, and consumer education	PENNY apprenticeship program (Germany) [49]
			Zero-waste restaurant (Portugal) [50]
	Coaching for households	Interventions aiming to reduce FW in households by improving food management skills through workshops, thematic challenges, personalized guidance, and community networks, focusing on planning, shopping, cooking, and storage practices	Cooking classes and workshops (Germany) [51]
			Tailored intervention with personalized coaching (USA) [52]
Awareness raising	Local initiatives	Community-focused interventions aiming to reduce FW through door-to-door visits, cooking workshops, awareness campaigns, and school or business engagement, emphasizing in-person interaction and collaboration with local stakeholders	Reduce FW, save money (Canada) [53]
			West London FW prevention campaign (United Kingdom) [54]
	Large-scale initiatives	Interventions take the form of broad awareness campaigns aimed at reducing FW by promoting behavior change through tools such as media outreach, exhibitions, and partnerships with retailers	FW-free week (Netherlands) [55]
			Great taste, no waste (United Kingdom) [56]
National programs		Large-scale interventions raising awareness of FW through media campaigns, educational materials, and stakeholder collaboration, promoting sustainable practices and systemic impacts by fostering partnerships and regional initiatives	Project Wasteless (Hungary) [57]
			Life FOODprint (Cyprus) [58]
Interventions uncovering new drivers		Interventions identifying new drivers of FW and testing innovative approaches, focusing on behaviors like overprovision during special occasions and poor planning, highlighting cultural contexts and social interactions	Education and leveraging social influence in school environments (Italy) [59]
			Study on domestic food practices (Italy) [60]
Out of scope	Measurement	Interventions aim to track and reduce surplus food through measurement or redistribute it to consumers and charities, minimizing FW via apps or food banks	Gladsaxe measurement (Denmark) [61]
			Copenhagen municipality (Denmark) [62]
	Redistribution		Olio app (51 countries globally) [63,64]
			Munch app (Hungary, Czech Republic, Slovakia, Romania) [65]

**Table 4:** Classification of FW prevention actions implemented by food services and households (based on [66]).

Type	Sub-Type	Description
FOOD SERVICES		
Supply chain efficiency	Process innovation	Innovating processes within food service establishments to increase efficiency and reduce waste. This includes implementing new technologies and improving current practices related to food handling and storage
	Training and guidelines	Providing training and guidelines for food service personnel to reduce FW, focusing on areas such as inventory management, portion control, and food preparation. This includes internal personnel training sessions and the development of best practice guides
	Public procurement	Integrating FW prevention criteria into public procurement processes for food services. This can include specifying requirements for waste reduction practices, such as sourcing locally to reduce transport losses and adopting sustainable food service practices
Consumer behavior change	Awareness/educational campaigns	Implementing campaigns to educate and raise awareness among consumers and personnel about the importance of reducing FW. This includes digital tools, school programs, and public campaigns aimed at changing FW behaviors
Food redistribution	Surplus food redistribution	Redistributing surplus food to charities or other organizations to ensure it is consumed rather than wasted. This includes collaboration with local food banks and other non-profits to handle excess food.
FW prevention governance	Voluntary agreement	Establishing voluntary agreements among stakeholders within the food service industry to commit to reducing FW. The agreements often involve setting shared goals, monitoring progress, and reporting on outcomes to ensure collective action toward FW reduction
	Regulatory framework/policy	Developing and implementing regulatory frameworks or policies that mandate FW reduction practices within the food service industry. This includes requirements for waste tracking, targets for waste reduction, and incentives for compliance
	National FW prevention program	Coordinating national programs that involve multiple stakeholders from the food service industry, the government, and non-profits to implement comprehensive strategies for FW prevention. This includes public awareness campaigns, support for innovation, and funding for waste reduction initiatives
HOUSEHOLDS		
Consumer behavior change	Awareness/educational campaigns	Launching educational initiatives to inform consumers about FW and provide practical tips for reducing waste at the household level. This includes workshops, digital tools, and media campaigns focused on planning food purchases, proper storage, and utilizing leftovers
	School programs	Implementing educational programs in schools to teach students about FW and encourage waste reduction behaviors that they can practice at home. The programs aim to build a culture of waste reduction from a young age
	Digital tools for behavioral change	Developing and promoting apps and online platforms that provide consumers with tips and strategies for reducing FW, tracking their food consumption, and planning meals more effectively. This includes mobile apps that remind users of expiration dates and suggest recipes based on available ingredients
	Innovation of products—date marking	Implementing initiatives to improve date marking on food products to help consumers better understand “best before” and “use by” dates. Examples include the introduction of labels such as “Best before … often good after” to encourage consumers to use their judgment before discarding food
Supply chain efficiency	Innovation of products—packaging	Innovating packaging solutions to extend the shelf life of food products, thus reducing the likelihood of food spoilage and waste at the household level. This includes creating more effective and sustainable packaging materials and technologies
FW prevention governance	Voluntary agreement	Establishing voluntary agreements among various stakeholders, including consumers, retailers, and local authorities to commit to reducing FW. The agreements involve setting targets, monitoring progress, and reporting outcomes to ensure collective action toward FW reduction
	National FW prevention program	Coordinating national programs that involve multiple stakeholders from households, the government, and non-profits to implement comprehensive strategies for FW prevention. The programs typically include public awareness campaigns, support for innovation, and funding for waste reduction initiatives

**Table 5:** FW-preventing interventions addressing students’ behavioral change.

Type of Intervention	Category and Description	Examples
Visual nudging interventions	Awareness raising: Implementing campaigns and visual tools to raise students’ awareness about FW issues and providing tips to adopt less wasteful behavior. The interventions often involve visual aids and the strategic placement of information to influence students’ decisions.	Using posters and signage to provide students with detailed information about the negative environmental, economic, and social impacts of FW [14,15,67,68,69,70,71] Displaying strategically placed posters and signs that encourage students to take only the food they intend to eat [72] Displaying posters that evoke negative social emotions associated with wasting food to discourage wasteful behavior [73] Placing visual reminders such as table talkers in dining areas to inform students about healthy food choices and/or the negative impact of FW [74,75] Utilizing posters highlighting social norms and peer behaviors regarding FW reduction to influence student choices [76]
Participatory nudging interventions	Interactive activities: Engaging students in hands-on, practical, or interactive experiences and competitive events focused on reducing FW. The activities encourage active participation and often involve a peer-driven approach to behavior change.	Involving students in FW audits to assess the amount of waste generated and identify key areas for improvement [14,77] Involving students in menu planning to make them more likely to adopt and advocate for waste-reducing behaviors [78,79] Organizing interactive activities where students participate in FW reduction challenges or competitions, making them more conscious about the amount of food they waste [14,80,81] Encouraging students to lead campaigns and create content (e.g., videos, posters) about FW, fostering a peer-driven approach to behavior change [71] Organizing food cooking workshops in school canteens to gain students’ practical skills and a better understanding of how their choices impact FW, promoting more sustainable behaviors [14,82] Installing digital bulletin boards with interactive content that educates students about FW and encourages them to take quizzes or participate in games related to food sustainability [83] Introducing mobile apps that allow students to track their FW and receive personalized tips and goals for reducing waste [83] Involving students in “food rescue programs” where leftover untouched food is collected and donated to local shelters or food banks, teaching them about food redistribution and community support [84,85] Installing interactive digital displays and touch-screen kiosks with quizzes and games related to food sustainability [86] Using color-coded waste bins with clear signage to guide students in sorting their waste correctly, making them more aware of how much food is being wasted [87]
Educational nudging interventions	Educational activities: Promoting responsible food consumption through various pedagogical approaches designed to foster long-term behavior change among students. The interventions focus on integrating food sustainability education into the curriculum and extra-curricular activities.	Educating students about the entire food system, from production to consumption, and deepening their understanding of and personal commitment to reducing FW [14,88,89,90] Developing a comprehensive curriculum that includes lessons, discussions, and assessments focused on FW and sustainability, ensuring that students encounter the topics across various subjects [14,68,90,91] Inviting guest speakers such as local farmers, chefs, or environmentalists to talk about food sustainability and waste reduction, providing real-world insights and inspiration [81] Organizing field trips to farms, food processing facilities, or waste management centers to give students a first-hand understanding of the food production and waste process [92,93]
Food choice architecture	Designing food choices: Designing and presenting food choices in a way that subtly influences students to select and consume their food more efficiently. This can involve the strategic placement and presentation of food items to promote healthier and less wasteful choices.	Allowing students to choose their food items rather than being served pre-determined portions ensures they take only what they plan to eat [14,94,95] Positioning items that are commonly wasted in more prominent locations, for example, placing vegetables at the beginning of the serving line so students are more likely to take and consume them [96,97,98,99,100] Offering fruits and vegetables in pre-sliced, ready-to-eat portions to encourage students to finish their servings, as these are more convenient and appealing than whole items [98,100] Using attractive names and presentations for healthier food options to make them more enticing [98,100,101] Introducing themed food days that focus on specific types of food (e.g., “Veggie Day”) to highlight and promote the consumption of particular food groups, reducing waste of those items [102,103,104] Organizing taste test events where students can sample small portions of different foods (e.g., “tasting spoons”) before deciding on their meal, reducing the likelihood of taking larger portions they might not finish [74,82]
Environment altering	Dining environment changes: Altering the dining environment and its conditions to encourage students to make sustainable food choices and practice responsible food consumption. The changes aim to create a more pleasant and quiet dining experience.	Extending the duration of lunch breaks to provide more time for students to eat slowly, thereby reducing FW and fostering responsible consumption [105,106,107] Changing plate sizes and shapes: when students serve themselves, offering smaller plates and different shapes can promote serving smaller portion sizes, thereby reducing the likelihood of plate waste; however, if the food is pre-served, using larger size plates is beneficial, as it allows students to clearly see and understand the ingredients of the food being served (more engaging for younger students) [27,108,109] Altering dining spaces by improving lighting, reducing noise levels, adding comfortable seating, and creating a pleasant atmosphere can make the dining experience more enjoyable, thereby encouraging students to appreciate and finish their meals [101,110] Establishing school gardens where students can grow their own fruits and vegetables and then use these in the school canteen, thus creating a direct connection between growing and consuming food [111,112]
Continuous improvement through feedback	Feedback and iteration: Regularly gathering feedback and insights from students on their food-wasting behavior and food preferences to refine and improve the interventions. This approach involves iterative processes to continuously enhance strategies based on collected data.	Implementing feedback systems where students can report on their FW habits and suggest improvements, potentially using digital tools to collect this feedback and iterating on strategies based on the data collected [42,113] Providing real-time feedback on FW levels in the canteen, such as through charts or digital displays, to raise students’ awareness about the environmental impact of their FW and to set goals to reduce it, thus allowing students to track progress and adjust their behavior accordingly [74,114,115,116] Regularly conducting surveys and polls to gather student opinions on menu items and dining experiences, using these data to make informed adjustments to the menu and dining environment [77,117,118]

**Table 6:** A unified menu designed for the field study.

	Placed Portion Planned Weight in Grams	Weight of Food Served on a Plate, Grams		Placed Portion Planned Weight, Grams	Weight of Food Served on a Plate, Grams
Monday			Thursday		
Pasta	130	130	Borscht (beet soup) with fresh cabbage and sour cream	200/5 *	205
Pork goulash	50/50	100	Chicken cutlet	80	80
Pickled cucumber	25	25	Stewed rice with carrots and corn	100/5	105
Cinnamon roll	1 piece	60	Fresh cucumber	30	30
Bread	25	25	Bread	25	25
Tuesday			Friday		
Rice soup with chicken meat and sour cream	150/22/5 *	177	Fish in breadcrumbs	60–65	63
Pork cutlet	50	50	Mashed potatoes	130	130
Mashed potatoes	130	130			
Fresh tomato salad with oil	50	50	Carrot salad with sunflower seeds	50	50
Bread (optional)	25	25	Glazed curd cheese	1 piece	46
Banana	1 piece	175 **	Bread	25	25
Wednesday					
Pork chop	60	60			
Mashed potatoes	130	130			
Fresh cabbage salad with carrots	50	50			
Bread	25	25			
Cupcake	50	50			
Apple	1 piece	140 **			

* Note: 5 g of sour cream is put in a common pot of soup, providing 5 g per 1 child. The weight of soup in each plate is 250 g. ** Note: average weight of 1 piece of banana and apple.

**Table 7:** Data on the amount of food served and total PW registered.

	September 2023				December 2023				April 2024			
School	Food Served, Total g	PW, Total g	PW, g per Stud. *	PW, % of the Food Served	Food Served, Total g	PW, Total g	PW, g per Stud.	PW, % of the Food Served	Food Served, Total g	PW, Total g	PW, g per Stud.	PW, % of the Food Served
S1	639,522	150,959	93	23.6	619,794	130,261	79	21.0	590,944	121,851	79	20.6
S2	587,279	129,006	90	22.0	577,767	117,347	78	20.3	593,915	117,555	82	19.8
S3	525,541	76,427	59	14.5	507,082	73,643	59	14.5	543,887	78,057	59	14.4
S4	653,534	152,481	108	23.3	627,717	109,991	81	17.5	615,617	131,764	99	21.4
Total	2,405,876	508,873	88	21.2	2,332,359	431,242	75	18.5	2,344,363	449,227	80	19.2

* Based on the actual number of students who participated in lunch breaks.

**Table 8:** Filtered data on food served and PW.

School	September 2023				December 2023				April 2024			
	Food Served, Total g	PW, Total g	PW, % of the Food Served	PW, g per Stud.	Food Served, Total g	PW, Total g	PW, % of the Food Served	PW, g per Stud.	Food Served, Total g	PW, Total g	PW, % of the Food Served	PW, g per Stud.
S1	475,057	90,922	19.1	56	475,666	76,428	16.1	47	450,681	80,395	17.8	52
S2	458,281	82,240	17.9	57	452,556	79,467	17.6	53	465,013	80,052	17.2	56
S3	363,520	43,679	12.0	34	356,514	53,644	15.0	43	377,378	57,233	15.2	43
S4	426,388	62,883	14.7	45	415,085	52,770	12.7	39	402,718	57,723	14.3	43
Total	1,723,245	279,723	16.2	48	1,699,821	262,309	15.4	46	1,695,790	275,403	16.2	49

**Table 9:** Data on surplus portions served.

	S1	S2	S3	S4
September 2023	1	133	0	103
December 2023	0	60	0	97
April 2024	0	182	0	78
Total	1	375	0	278

**Table 10:** Data on filtered PW in the short run, g/student, September 2023–December 2023 (class view).

Grades	S1		S2		S3		S4	
	Sept.	Dec.	Sept.	Dec.	Sept.	Dec.	Sept.	Dec.
1a	59	51	53	64	29	50	64	45
1b	63	53	61	63	33	33	33	39
1c	x	x	84	72	x	x	x	x
2a	67	53	63	68	32	49	42	31
2b	62	57	56	58	58	56	59	45
2c	x	x	65	56	x	x	x	x
3a	48	38	47	56	32	52	57	49
3b	75	56	44	44	38	37	52	39
3c	x	x	53	54	x	x	x	x
4a	53	49	64	45	22	38	39	39
4b	55	46	54	56	52	39	44	49
4c	x	x	x	x	58	56	x	x
5a	59	35	57	28	32	54	56	38
5b	63	55	73	62	12	23	35	20
5c	39	32	x	x	x	x	46	49
6a	49	38	62	51	36	41	30	32
6b	53	40	58	57	42	52	38	40
7a	42	45	50	44	28	40	49	25
7b	45	40	26	22	15	44	25	36
7c	x	x	x	x	19	22	x	x
	*p*-value = 0.000		*p*-value = 0.159		*p*-value = 0.011		*p*-value = 0.095	

**Table 11:** Data on filtered PW in the long run, g/student, September 2023–April 2024 (class view).

Grades	S1		S2		S3		S4	
	Sept.	Apr.	Sept.	Apr.	Sept.	Apr.	Sept.	Apr.
1a	59	52	53	53	29	39	64	56
1b	63	74	61	66	33	25	33	23
1c	x	x	84	91	x	x	x	x
2a	67	58	63	76	32	48	42	34
2b	62	67	56	51	58	52	59	54
2c	x	x	65	69	x	x	x	x
3a	48	56	47	67	32	46	57	56
3b	75	51	44	55	38	43	52	61
3c	x	x	53	51	x	x	x	x
4a	53	54	64	44	22	31	39	30
4b	55	52	54	68	52	64	44	39
4c	x	x	x	x	58	74	x	x
5a	59	50	57	34	32	43	56	32
5b	63	52	73	59	12	34	35	32
5c	39	28	x	x	x	x	46	69
6a	49	40	62	43	36	38	30	40
6b	53	41	58	71	42	63	38	46
7a	42	52	50	48	28	39	49	34
7b	45	39	26	27	15	36	25	40
7c	x	x	x	x	19	9	x	x
	*p*-value = 0.107		*p*-value = 0.890		*p*-value = 0.004		*p*-value = 0.639	

**Table 12:** Data on filtered PW in the short run, g/student, September 2023–December 2023 (day view).

	S1		S2		S3		S4	
	Sept.	Dec.	Sept.	Dec.	Sept.	Dec.	Sept.	Dec.
Monday	49.83	47.49	47.24	39.94	21.35	35.22	29.08	37.43
Tuesday	31.97	39.92	50.94	43.64	24.26	31.18	40.49	34.56
Wednesday	49.19	44.76	55.59	48.74	33.65	33.92	49.36	44.80
Thursday	94.50	60.29	84.97	72.67	51.21	79.22	53.59	42.70
Friday	53.62	40.36	52.79	58.19	36.98	32.98	50.04	35.20
	*p*-value = 0.313		*p*-value = 0.125		*p*-value = 0.188		*p*-value = 0.313	

**Table 13:** Data on filtered PW in the long run, g/student, 23 September–24 April (day view).

	S1		S2		S3		S4	
	Sept.	Apr.	Sept.	Apr.	Sept.	Apr.	Sept.	Apr.
Monday	49.83	41.06	47.24	47.93	21.35	34.65	29.08	39.22
Tuesday	31.97	55.52	50.94	63.32	24.26	45.83	40.49	41.12
Wednesday	49.19	51.80	55.59	56.02	33.65	36.10	49.36	44.85
Thursday	94.50	66.70	84.97	62.32	51.21	62.03	53.59	49.71
Friday	53.62	46.53	52.79	52.27	36.98	38.10	50.04	41.05
	*p*-value = 0.813		*p*-value = 1.000		*p*-value = 0.063		*p*-value = 0.813	

## Data Availability

The original contributions presented in the study are included in the article, further inquiries can be directed to the corresponding author.

## References

[1] United Nations General Assembly (2015). Transforming Our World: The 2030 Agenda for Sustainable Development.

[2] FAO (2011). Global Food Losses and Food Waste—Extent, Causes and Prevention.

[3] Cuéllar A.D., Webber M.E. (2010). Wasted Food, Wasted Energy: The Embedded Energy in Food Waste in the United States. Environ. Sci. Technol..

[4] Kummu M., de Moel H., Porkka M., Siebert S., Varis O., Ward P.J. (2012). Lost Food, Wasted Resources: Global Food Supply Chain Losses and Their Impacts on Freshwater, Cropland, and Fertiliser Use. Sci. Total Environ..

[5] Godfray H.C.J., Beddington J.R., Crute I.R., Haddad L., Lawrence D., Muir J.F., Pretty J., Robinson S., Thomas S.M., Toulmin C. (2010). Food Security: The Challenge of Feeding 9 Billion People. Science.

[6] Wunder S. (2018). Food Waste Prevention and Valorisation: Relevant EU Policy Areas. REFRESH Deliverable 3.3. https://eu-refresh.org/sites/default/files/REFRESH_D3.3_EU%20policy%20screening_18052018_25072018.pdf.

[7] United Nations Environment Programme (2024). Food Waste Index Report 2024. Think Eat Save: Tracking Progress to Halve Global Food Waste.

[8] Eurostat Home Page. https://ec.europa.eu/eurostat/statistics-explained/index.php?title=Food_waste_and_food_waste_prevention_-_estimates.

[9] European Commission (2023). Commission Staff Working Document: Impact Assessment Report Accompanying the Document Directive of the European Parliament and of the Council Amending Directive 2008/98/EC on Waste.

[10] European Commission (2018). Communication from the Commission to the European Parliament, the Council, the European Economic and Social Committee, and the Committee of the Regions: A Sustainable Bioeconomy for Europe: Strengthening the Connection Between Economy, Society and the Environment.

[11] European Commission (2020). Communication from the Commission to the European Parliament, the Council, the European Economic and Social Committee, and the Committee of the Regions: A New Circular Economy Action Plan. For a Cleaner and More Competitive Europe 2020.

[12] European Commission (2020). Communication from the Commission to the European Parliament, the Council, the European Economic and Social Committee, and the Committee of the Regions: A Farm to Fork Strategy for a Fair, Healthy and Environmentally-Friendly Food System.

[13] European Commission (2020). Communication from the Commission to the European Parliament, the Council, the European Economic and Social Committee, and the Committee of the Regions: EU Biodiversity Strategy for 2030. Bringing Nature Back into Our Lives.

[14] Derqui B., Fernandez V., Fayos T. (2018). Towards More Sustainable Food Systems. Addressing Food Waste at School Canteens. Appetite.

[15] Prescott M.P., Burg X., Metcalfe J.J., Lipka A.E., Herritt C., Cunningham-Sabo L. (2019). Healthy Planet, Healthy Youth: A Food Systems Education and Promotion Intervention to Improve Adolescent Diet Quality and Reduce Food Waste. Nutrients.

[16] Kaur P., Dhir A., Talwar S., Alrasheedy M. (2021). Systematic Literature Review of Food Waste in Educational Institutions: Setting the Research Agenda. Int. J. Contemp. Hosp. Manag..

[17] Jeswani H.K., Figueroa-Torres G., Azapagic A. (2021). The Extent of Food Waste Generation in the UK and Its Environmental Impacts. Sustain. Prod. Consum..

[18] Stenmarck Å., Jensen C., Quested T., Moates G. (2016). Estimates of European Food Waste Levels.

[19] Wunder S. (2019). REFRESH Policy Brief: Reducing Consumer Food Waste. https://www.ecologic.eu/16393.

[20] European Commission Home Page. The European Green Deal Striving to be the First Climate-Neutral Continent. https://commission.europa.eu/strategy-and-policy/priorities-2019-2024/european-green-deal_en.

[21] European Commission: Directorate-General for Research and Innovation and Group of Chief Scientific Advisors (2020). Towards a Sustainable Food System—Moving from Food as a Commodity to Food as More of a Common Good—Independent Expert Report.

[22] Fabbri K., Froidmont-Görtz I., Faure U., Ndongosi I., Gajdzinska M., Haentjens W., Krommer J., Lizaso M., Lutzeyer H.-J., Fabbri K., Ndongosi I., European Commission: Directorate-General for Research and Innovation (2020). Food 2030 Pathways for Action—Research and Innovation Policy as a Driver for Sustainable, Healthy and Inclusive Food Systems.

[23] United Nations Environment Programme (2021). Food Waste Index Report 2021.

[24] Getts K.M., Quinn E.L., Johnson D.B., Otten J.J. (2017). Validity and Interrater Reliability of the Visual Quarter-Waste Method for Assessing Food Waste in Middle School and High School Cafeteria Settings. J. Acad. Nutr. Diet..

[25] European Environment Agency Home Page. https://www.eea.europa.eu/themes/waste/waste-prevention/countries.

[26] Āriņa D., Teibe I., Bendere R., Melnalksne Z. Food Waste Estimation in Latvia. Proceedings of the 9th International Conference on Sustainable Solid Waste Management.

[27] Lonska J., Zvaigzne A., Kotane I., Silicka I., Litavniece L., Kodors S., Deksne J., Vonoga A. (2022). Plate Waste in School Catering in Rezekne, Latvia. Sustainability.

[28] Quested T.E., Marsh E., Stunell D., Parry A.D. (2013). Spaghetti Soup: The Complex World of Food Waste Behaviours. Resour. Conserv. Recycl..

[29] van Geffen L., van Herpen E., van Trijp H. (2016). Causes & Determinants of Consumers Food Waste. REFRESH Deliverable 1.1.

[30] Ölander F., Thøgersen J. (1995). Understanding of Consumer Behaviour as a Prerequisite for Environmental Protection. J Consum Policy.

[31] Rothschild M.L. (1999). Carrots, Sticks, and Promises: A Conceptual Framework for the Management of Public Health and Social Issue Behaviors. J. Mark..

[32] Davison S., van Geffen L., van Herpen E., Sharp A. (2020). Applying Behaviour Change Methods to Food Waste. Routledge Handbook of Food Waste.

[33] National Academies of Sciences, Engineering, and Medicine (2020). A National Strategy to Reduce Food Waste at the Consumer Level.

[34] van Geffen L., van Herpen E., Sijtsema S., van Trijp H. (2020). Food Waste as the Consequence of Competing Motivations, Lack of Opportunities, and Insufficient Abilities. Resour. Conserv. Recycl. X.

[35] Vittuari M., Garcia Herrero L., Masotti M., Iori E., Caldeira C., Qian Z., Bruns H., van Herpen E., Obersteiner G., Kaptan G. (2023). How to Reduce Consumer Food Waste at Household Level: A Literature Review on Drivers and Levers for Behavioural Change. Sustain. Prod. Consum..

[36] Wunder S., van Herpen E., McFarland K., Ritter A., van Geffen L., Stenmarck Å., Hulten J. (2019). Policies Against Consumer Food Waste. Policy Options for Behaviour Change Including Public Campaigns. REFRESH Deliverable 3.4. https://eu-refresh.org/policies-against-consumer-food-waste.html.

[37] European Commission Home Page Supporting Policy with Scientific Evidence. https://knowledge4policy.ec.europa.eu/projects-activities/european-consumer-food-waste-forum_en.

[38] Swannell R., Bruns H., Brüggemann N., Candeal T., Casonato C., Diercxsens C., Garcia Herrero L., Gil Roig J.M., Haglund Y., van Herpen E. (2023). Evaluation of Consumer Food Waste Prevention Interventions.

[39] University of Exeter Home Page. https://www.exeter.ac.uk/research/foodt/.

[40] OzHarvest (2022). Use It Up Tape™. Impact Study Reducing Household Food Waste by 40%. https://www.ozharvest.org/app/uploads/2023/05/OzHarvest-Use-It-Up-Tape-Impact-Study.pdf.

[41] Lessfoodwaste Home Page. https://www.lessfoodwaste.fi/paulig/en/Home.

[42] Lim V., Bartram L., Funk M., Rauterberg M. (2021). Eco-Feedback for Food Waste Reduction in a Student Residence. Front. Sustain. Food Syst..

[43] The Waste and Resources Action Programme (WRAP) Home Page. https://www.wrap.ngo/resources/case-study/behaviour-change-intervention-day-date-label.

[44] Too Good to Go Home Page. https://www.toogoodtogo.com/en-ca/look-smell-taste.

[45] Vidal-Mones B., Diaz-Ruiz R., Gil J.M. (2022). From Evaluation to Action: Testing Nudging Strategies to Prevent Food Waste in School Canteens. Waste Manag..

[46] International Food Waste Coalition (2022). Engaging with Consumers to Cut Food Waste. Findings from an Experiment Using Social Norm Interventions to Reduce Plate Waste in Corporate Restaurant.

[47] Wageningen University & Research Home Page. https://www.wur.nl/en/project/effectiveness-smaaklessen.htm.

[48] Decojovem Home Page. https://decojovem.pt/pt/recursos/projeto/green-chef.

[49] EU Refresh Home Page. https://eu-refresh.org/food-waste-reduction-training-penny-apprentices-successfully-completed.html.

[50] PPL Home Page. https://ppl.pt/en/kitchen-dates.

[51] Slow Food Deutschland (and Technical University of Berlin) Küchenlabore im Dialogforum “Private Haushalte”. https://www.zugutfuerdietonne.de/fileadmin/zgfdt/sektorspezifische_Dialogforen/Private_Haushalte/01.12.22/K%C3%BCchenlabore_im_Dialogforum_private_Haushalte_-_Bericht.pdf.

[52] Roe B.E., Qi D., Beyl R.A., Neubig K.E., Apolzan J.W., Martin C.K. (2022). A Randomized Controlled Trial to Address Consumer Food Waste with a Technology-Aided Tailored Sustainability Intervention. Resour. Conserv. Recycl..

[53] van der Werf P., Seabrook J.A., Gilliland J.A. (2021). “Reduce Food Waste, Save Money”: Testing a Novel Intervention to Reduce Household Food Waste. Environ. Behav..

[54] Quested T.E., Ingle R., Waste Resources and Action Programme (WRAP) (2013). West London Food Waste Prevention Campaign Evaluation Report.

[55] Samen Tegen Voedselverspilling Home Page. https://samentegenvoedselverspilling.nl/en.

[56] Vaisanen I. The Icon Home Page. https://www.theicon.org.uk/lidl-and-love-food-hate-waste-scotland-team-up-to-tackle-food-waste/.

[57] Maradeknelkul Home Page. https://maradeknelkul.hu/en/about-wasteless/.

[58] Food Print (2023). Life FOODprint: Awareness–Raising Campaign to Prevent and Manage Food Waste Among Consumers and the Food and Hospitality Industries. Consultation Report.

[59] Piras S., Righi S., Banchelli F., Giordano C., Setti M. (2023). Food Waste between Environmental Education, Peers, and Family Influence. Insights from Primary School Students in Northern Italy. J. Clean. Prod..

[60] Romani S., Grappi S., Bagozzi R.P., Barone A.M. (2018). Domestic Food Practices: A Study of Food Management Behaviors and the Role of Food Preparation Planning in Reducing Waste. Appetite.

[61] Gladsaxe Municipality (2023). Voluntary Local Review from Gladsaxe 2023.

[62] Vrzel J., Circular Innovation Lab (2022). Food Waste in the Municipality of Copenhagen: A Circular Economy Vision.

[63] Makov T., Shepon A., Krones J., Gupta C., Chertow M. (2020). Social and Environmental Analysis of Food Waste Abatement via the Peer-to-Peer Sharing Economy. Nat. Commun..

[64] Olio App Home page. https://olioapp.com/en/getting-started-on-olio/what-is-olio/.

[65] Munch Home Page. https://munch.eco/.

[66] Caldeira C., De Laurentiis V., Sala S. (2019). Assessment of Food Waste Prevention Actions: Development of an Evaluation Framework to Assess Performance of Food Waste Prevention Actions.

[67] Qian L., Zhao X., Liu G. (2024). The Association between the Awareness Campaign and Food Waste among University Students in China. Resour. Conserv. Recycl..

[68] Antón-Peset A., Fernandez-Zamudio M.-A., Pina T. (2021). Promoting Food Waste Reduction at Primary Schools. A Case Study. Sustainability.

[69] European Commission (2023). Reducing Consumer Food Waste: Recommendations for Schools.

[70] Ozcicek-Dolekoglu C., Var I. (2019). Analysis of food waste in university dining halls: A case study from Turkey. Fresenius Environ. Bull..

[71] Pinto R.M.d.S., Melo F.F.S., Campos S.S., Cordovil C.M.-S. (2018). A Simple Awareness Campaign to Promote Food Waste Reduction in a University Canteen. Waste Manag..

[72] Kirshnan A., Luna J., Voronkova K., Huang V., Hernandez D., Batth J. (2024). The Effects of Motivational and Informative Signage on Intentions to Minimize Food Waste in a University All-Access Dining Hall.

[73] Sutinen U.M., Närvänen E., Mesiranta N., Mattila M., Heikkinen A. (2020). Assumptions About Consumers in Food Waste Campaigns: A Visual Analysis. Food Waste Management: Solving the Wicked Problem.

[74] Malefors C., Sundin N., Tromp M., Eriksson M. (2022). Testing Interventions to Reduce Food Waste in School Catering. Resour. Conserv. Recycl..

[75] Malefors C. (2022). Food Waste Reduction in the Public Catering Sector. Ph.D. Thesis.

[76] Yamin P., Fei M., Lahlou S., Levy S. (2019). Using Social Norms to Change Behavior and Increase Sustainability in the Real World: A Systematic Review of the Literature. Sustainability.

[77] Wyse R., Jackson J., Stacey F., Delaney T., Ivers A., Lecathelinais C., Sutherland R. (2022). The Effectiveness of Canteen Manager Audit and Feedback Reports and Online Menu-Labels in Encouraging Healthier Food Choices within Students’ Online Lunch Orders: A Pilot Cluster Randomised Controlled Trial in Primary School Canteens in New South Wales, Australia. Appetite.

[78] Dekšne J., Litavniece L., Lonska J., Zvaigzne A. (2023). Circular Economy Strategies for Reducing Food Waste in Schools: A Systematic Literature Review. J. Reg. Econ. Soc. Dev..

[79] Pagliarino E., Santanera E., Falavigna G. (2021). Opportunities for and Limits to Cooperation between School and Families in Sustainable Public Food Procurement. Sustainability.

[80] Yen D.A., Cappellini B., Dovey T. (2022). Primary School Children’s Responses to Food Waste at School. Br. Food J..

[81] Rolker H., Eisler M., Cardenas L., Deeney M., Takahashi T. (2022). Food Waste Interventions in Low-and-Middle-Income Countries: A Systematic Literature Review. Resour. Conserv. Recycl..

[82] Serebrennikov D., Katare B., Kirkham L., Schmitt S. (2020). Effect of Classroom Intervention on Student Food Selection and Plate Waste: Evidence from a Randomized Control Trial. PLoS ONE.

[83] Yu Y., Yi S., Nan X., Lo L.Y., Shigyo K., Xie L., Wicaksana J., Cheng K.T., Qu H. (2023). FoodWise: Food Waste Reduction and Behavior Change on Campus with Data Visualization and Gamification. Proceedings of the 6th ACM SIGCAS/SIGCHI Conference on Computing and Sustainable Societies (COMPASS’23), Cape Town, South Africa, 16–19 August 2023.

[84] Schupp C.L., Getts K.M., Otten J.J. (2018). An Evaluation of Current Lunchroom Food Waste and Food Rescue Programs in a Washington State School District. J. Agric. Food Syst. Community Dev..

[85] Hecht A.A., Neff R.A. (2019). Food Rescue Intervention Evaluations: A Systematic Review. Sustainability.

[86] Soma T., Li B., Maclaren V. (2020). Food Waste Reduction: A Test of Three Consumer Awareness Interventions. Sustainability.

[87] Leeabai N., Areeprasert C., Khaobang C., Viriyapanitchakij N., Bussa B., Dilinazi D., Takahashi F. (2021). The Effects of Color Preference and Noticeability of Trash Bins on Waste Collection Performance and Waste-Sorting Behaviors. Waste Manag..

[88] Chu C.-M., Chih C., Teng C.-C. (2023). Food Waste Management: A Case of Taiwanese High School Food Catering Service. Sustainability.

[89] Ellison B., Savchenko O., Nikolaus C.J., Duff B.R.L. (2019). Every Plate Counts: Evaluation of a Food Waste Reduction Campaign in a University Dining Hall. Resour. Conserv. Recycl..

[90] Pierre C.S., Sokalsky A., Sacheck J.M. (2024). Participant Perspectives on the Impact of a School-Based, Experiential Food Education Program Across Childhood, Adolescence, and Young Adulthood. J. Nutr. Educ. Behav..

[91] Derqui B., Fernandez V. (2017). The Opportunity of Tracking Food Waste in School Canteens: Guidelines for Self-Assessment. Waste Manag..

[92] 4 Roots Farm Home page. https://4rootsfarm.org/our-programs/fieldtrips/.

[93] Food Print Home Page. https://foodprint.org/issues/farm-to-school-and-garden-education/.

[94] Chang Y.Y.-C. (2022). All You Can Eat or All You Can Waste? Effects of Alternate Serving Styles and Inducements on Food Waste in Buffet Restaurants. Curr. Issues Tour..

[95] Liu Y., Cheng S., Liu X., Cao X., Xue L., Liu G. (2016). Plate Waste in School Lunch Programs in Beijing, China. Sustainability.

[96] Adams M.A., Bruening M., Ohri-Vachaspati P., Hurley J.C. (2016). Location of School Lunch Salad Bars and Fruit and Vegetable Consumption in Middle Schools: A Cross-Sectional Plate Waste Study. J. Acad. Nutr. Diet..

[97] Garnett E.E., Marteau T.M., Sandbrook C., Pilling M.A., Balmford A. (2020). Order of Meals at the Counter and Distance between Options Affect Student Cafeteria Vegetarian Sales. Nat. Food.

[98] Greene K.N., Gabrielyan G., Just D.R., Wansink B. (2017). Fruit-Promoting Smarter Lunchrooms Interventions: Results From a Cluster RCT. Am. J. Prev. Med..

[99] Cohen J.F.W., Richardson S.A., Cluggish S.A., Parker E., Catalano P.J., Rimm E.B. (2015). Effects of Choice Architecture and Chef-Enhanced Meals on the Selection and Consumption of Healthier School Foods: A Randomized Clinical Trial. JAMA Pediatr..

[100] Thompson E., Johnson D.C., Leite-Bennett A., Ding Y., Mehrotra K. (2017). The Impact of Multiple Strategies to Encourage Fruit and Vegetable Consumption During School Lunch. J. Sch. Health.

[101] Elnakib S.A., Quick V., Mendez M., Downs S., Wackowski O.A., Robson M.G. (2021). Food Waste in Schools: A Pre-/Post-Test Study Design Examining the Impact of a Food Service Training Intervention to Reduce Food Waste. Int. J. Environ. Res. Public Health.

[102] Lorenz-Walther B.A.-S., Langen N. (2020). Sustainable Changes in a Worksite Canteen: An Exploratory Study on the Acceptance of Guests. J. Clean. Prod..

[103] Petruzzelli M., García-Herrero L., De Menna F., Vittuari M. (2023). Towards Sustainable School Meals: Integrating Environmental and Cost Implications for Nutritious Diets through Optimisation Modelling. Sustain. Sci..

[104] Gardner G., Burton W., Sinclair M., Bryant M. (2023). Interventions to Strengthen Environmental Sustainability of School Food Systems: Narrative Scoping Review. Int. J. Environ. Res. Public Health.

[105] Burton M., Wood J.M., Booth A.O., Worsley A., Larsson C., Margerison C. (2022). Enough Time for Lunch? The Duration and Governance of Lunch Eating Times in Australian Primary Schools: A Mixed-Methods Study. Appetite.

[106] Kodors S., Zvaigzne A., Litavniece L., Lonska J., Silicka I., Kotane I., Deksne J. (2022). Plate Waste Forecasting Using the Monte Carlo Method for Effective Decision Making in Latvian Schools. Nutrients.

[107] Qian L., Li F., Cao B., Wang L., Jin S. (2021). Determinants of Food Waste Generation in Chinese University Canteens: Evidence from 9192 University Students. Resour. Conserv. Recycl..

[108] Anderson S.M., Olds D.A., Wolfe K.L. (2021). The Impact of a Portion Plate on Plate Waste in a University Dining Hall. J. Foodserv. Manag. Educ..

[109] Ravandi B., Jovanovic N. (2019). Impact of Plate Size on Food Waste: Agent-Based Simulation of Food Consumption. Resour. Conserv. Recycl..

[110] Mumby S., Leineweber M., Andrade J. (2018). The Impact the Smarter Lunchroom Movement Strategies Have on School Children’s Healthy Food Selection and Consumption: A Systematic Review. J. Child. Nutr. Manag..

[111] Maliotou M.N., Liarakou G. (2014). School Gardening through the Perspective of the Whole School Approach. Education 3-13.

[112] Schreinemachers P., Ouedraogo M.S., Diagbouga S., Thiombiano A., Kouamé S.R., Sobgui C.M., Chen H.-P., Yang R.-Y. (2019). Impact of School Gardens and Complementary Nutrition Education in Burkina Faso. J. Dev. Eff..

[113] Santana S.A., Batista S.A., da Costa Maynard D., Ginani V.C., Zandonadi R.P., Botelho R.B.A. (2023). Acceptability of School Menus: A Systematic Review of Assessment Methods. Int. J. Environ. Res. Public Health.

[114] Malefors C., Svensson E., Eriksson M. (2024). Automated Quantification Tool to Monitor Plate Waste in School Canteens. Resour. Conserv. Recycl..

[115] Hanks A.S., Wansink B., Just D.R. (2014). Reliability and Accuracy of Real-Time Visualization Techniques for Measuring School Cafeteria Tray Waste: Validating the Quarter-Waste Method. J. Acad. Nutr. Diet..

[116] Koohmarey D., Nagesh N. (2019). Food Waste Estimation Using Received Signal Strength Indicator. arXiv.

[117] Elnakib S., Landry M.J., Farris A.R., Byker Shanks C. (2024). Strategies to Address Food Waste in K-12 Schools: A Narrative Review. J. Child Nutr. Manag..

[118] Eustachio Colombo P., Patterson E., Lindroos A.K., Parlesak A., Elinder L.S. (2020). Sustainable and Acceptable School Meals through Optimization Analysis: An Intervention Study. Nutr. J..

[119] Boschini M., Falasconi L., Cicatiello C., Franco S. (2020). Why the Waste? A Large-Scale Study on the Causes of Food Waste at School Canteens. J. Clean. Prod..

[120] Boulet M., Grant W., Hoek A., Raven R. (2022). Influencing across Multiple Levels: The Positive Effect of a School-Based Intervention on Food Waste and Household Behaviours. J. Environ. Manag..

[121] García-Herrero L., De Menna F., Vittuari M. (2019). Food Waste at School. The Environmental and Cost Impact of a Canteen Meal. Waste Manag..

[122] Lagorio A., Pinto R., Golini R. (2018). Food Waste Reduction in School Canteens: Evidence from an Italian Case. J. Clean. Prod..

[123] Liz Martins M., Rodrigues S.S.P., Cunha L.M., Rocha A. (2020). Factors Influencing Food Waste during Lunch of Fourth-Grade School Children. Waste Manag..

[124] Zvaigzne A., Litavniece L., Lonska J., Kodors S., Silicka I., Kotane I., Zukovs V., Gravite V., Amelcenkova L., Deksne J. (2021). Report on the Results of the Project “E-Mentor as a Transformation Tool for Ensuring Zero-Waste Food Consumption in Educational Institutions” and the Policy Recommendations.

[125] Betz A., Buchli J., Göbel C., Müller C. (2015). Food Waste in the Swiss Food Service Industry—Magnitude and Potential for Reduction. Waste Manag..

[126] Adams M.A., Pelletier R.L., Zive M.M., Sallis J.F. (2005). Salad Bars and Fruit and Vegetable Consumption in Elementary Schools: A Plate Waste Study. J. Am. Diet. Assoc..

[127] Buzby J.C., Guthrie J.F. (2022). Plate Waste in School Nutrition Programs: Final Report to Congress.

[128] Byker C.J., Farris A.R., Marcenelle M., Davis G.C., Serrano E.L. (2014). Food Waste in a School Nutrition Program After Implementation of New Lunch Program Guidelines. J. Nutr. Educ. Behav..

[129] Cohen J.F.W., Richardson S., Austin S.B., Economos C.D., Rimm E.B. (2013). School Lunch Waste Among Middle School Students: Nutrients Consumed and Costs. Am. J. Prev. Med..

[130] Engström R., Carlsson-Kanyama A. (2004). Food Losses in Food Service Institutions Examples from Sweden. Food Policy.

[131] Falasconi L., Vittuari M., Politano A., Segrè A. (2015). Food Waste in School Catering: An Italian Case Study. Sustainability.

[132] Marlette M.A., Templeton S.B., Panemangalore M. (2005). Food Type, Food Preparation, and Competitive Food Purchases Impact School Lunch Plate Waste by Sixth-Grade Students. J. Am. Diet. Assoc..

[133] Papargyropoulou E., Lozano R., Steinberger J.K., Wright N., Ujang Z. (2014). bin The Food Waste Hierarchy as a Framework for the Management of Food Surplus and Food Waste. J. Clean. Prod..

[134] LOWINFOOD Home Page. https://lowinfood.eu/2022/04/11/plate-waste-tracking-in-uppsala/.

[135] Yoon S.J., Kim H.A. (2012). Elementary School Students’ Perception of Food Waste and Factors Affecting Plate Waste Rate of School Foodservice in the Gyeongnam Area. J. Korean Diet. Assoc..

[136] Persson Osowski C., Osowski D., Johansson K., Sundin N., Malefors C., Eriksson M. (2022). From Old Habits to New Routines—A Case Study of Food Waste Generation and Reduction in Four Swedish Schools. Resources.

[137] Visschers V.H.M., Gundlach D., Beretta C. (2020). Smaller Servings vs. Information Provision: Results of Two Interventions to Reduce Plate Waste in Two University Canteens. Waste Manag..

[138] Food and Agriculture Organization of the United Nations (FAO) Home Page. https://www.fao.org/faolex/results/details/en/c/LEX-FAOC083580/.

[139] Candeal T., Brüggemann N., Bruns H., Casonato C., Diercxsens C., García-Herrero L., Gil J.M., Haglund Y., Kaptan G., Kasza G. (2023). Tools, Best Practices and Recommendations to Reduce Consumer Food Waste—A Compendium.

[140] Bustamente M., Afonso A., De los Ríos I. (2018). Exploratory Analysis of Food Waste at Plate in School Canteens in Spain. La Granja Rev. De Cienc. De La Vida.

[141] Tuorila H., Palmujoki I., Kytö E., Törnwall O., Vehkalahti K. (2015). School Meal Acceptance Depends on the Dish, Student, and Context. Food Qual. Prefer..

[142] Zhao X., Manning L. (2019). Food Plate Waste: Factors Influencing Insinuated Intention in a University Food Service Setting. Br. Food J..

[143] Blondin S.A., Djang H.C., Metayer N., Anzman-Frasca S., Economos C.D. (2015). ‘It’s Just so Much Waste.’ A Qualitative Investigation of Food Waste in a Universal Free School Breakfast Program. Public Health Nutr..

[144] Zandian M., Ioakimidis I., Bergström J., Brodin U., Bergh C., Leon M., Shield J., Södersten P. (2012). Children Eat Their School Lunch Too Quickly: An Exploratory Study of the Effect on Food Intake. BMC Public Health.

[145] Zhao C., Panizza C., Fox K., Boushey C.J., Byker Shanks C., Ahmed S., Chen S., Serrano E.L., Zee J., Fialkowski M.K. (2019). Plate Waste in School Lunch: Barriers, Motivators, and Perspectives of SNAP-Eligible Early Adolescents in the US. J. Nutr. Educ. Behav..

[146] Bschaden A., Dörsam A.F., Cvetko K., Kalamala T., Stroebele-Benschop N. (2020). The Impact of Lighting and Table Linen as Ambient Factors on Meal Intake and Taste Perception. Food Qual. Prefer..

[147] Park E.-S., Lee J.-H., Kim M.-H. (2015). Eating Habits and Food Preferences of Elementary School Students in Urban and Suburban Areas of Daejeon. Clin. Nutr. Res..

[148] Tóth A.J., Dunay A., Bálint Illés C., Battay M., Bittsánszky A., Süth M. (2023). Food Liking and Consumption in Schools: Comparison of Questionnaire-Based Surveys with Real Consumption. Food Qual. Prefer..

[149] Aires C., Saraiva C., Fontes M.C., Moreira D., Moura-Alves M., Gonçalves C. (2021). Food Waste and Qualitative Evaluation of Menus in Public University Canteens—Challenges and Opportunities. Foods.

[150] Cohen J.F.W., Smit L.A., Parker E., Austin S.B., Frazier A.L., Economos C.D., Rimm E.B. (2012). Long-Term Impact of a Chef on School Lunch Consumption: Findings from a 2-Year Pilot Study in Boston Middle Schools. J. Acad. Nutr. Diet..

[151] Moura D., Nunes C., Sancho T.S., Vidinha D. (2024). Assessment of Food Waste in Public Preschool and Primary Schools at the Municipality of Faro. Acta Port. Nutr..

[152] Carvalho J.G., Lima J.P.M., da Rocha A.M.C.N. (2015). Desperdício Alimentar E Satisfação Do Consumidor Com O Serviço De Alimentação Da Escola De Hotelaria E Turismo De Coimbra, Portugal. DEMETRA Aliment. Nutr. Saúde.

[153] Sehnem S., Godoi L., Simioni F., Martins C., Soares S.V., de Andrade Guerra J.B.S.O., Provensi T. (2023). Management Food Waste in Municipality Schools: An Analysis from a Circular Economy Perspective. Logistics.

[154] Favuzzi N., Trerotoli P., Forte M.G., Bartolomeo N., Serio G., Lagravinese D., Vino F. (2020). Evaluation of an Alimentary Education Intervention on School Canteen Waste at a Primary School in Bari, Italy. Int. J. Environ. Res. Public Health.

[155] Steen H., Malefors C., Röös E., Eriksson M. (2018). Identification and Modelling of Risk Factors for Food Waste Generation in School and Pre-School Catering Units. Waste Manag..

[156] Sundin N., Malefors C., Strotmann C., Orth D., Kaltenbrunner K., Obersteiner G., Scherhaufer S., Sjölund A., Persson Osowski C., Strid I. (2024). Sustainability Assessment of Educational Approaches as Food Waste Prevention Measures in School Catering. J. Clean. Prod..

[157] Liz Martins M., Rodrigues S.S., Cunha L.M., Rocha A. (2016). Strategies to Reduce Plate Waste in Primary Schools—Experimental Evaluation. Public Health Nutr..

[158] International Food Waste Coalition (2018). SKOOL2018 REPORT.

[159] Whitehair K.J., Shanklin C.W., Brannon L.A. (2013). Written Messages Improve Edible Food Waste Behaviors in a University Dining Facility. J. Acad. Nutr. Diet..

[160] Nisa C.F., Bélanger J.J., Schumpe B.M. (2022). Assessing the Effectiveness of Food Waste Messaging. Environ. Sci. Policy.

[161] Richardson R., Prescott M.P., Ellison B. (2021). Impact of Plate Shape and Size on Individual Food Waste in a University Dining Hall. Resour. Conserv. Recycl..

